# An Evaluation of Function of Multicopy Noncoding RNAs in Mammals Using ENCODE/FANTOM Data and Comparative Genomics

**DOI:** 10.1093/molbev/msy046

**Published:** 2018-04-03

**Authors:** Marc P Hoeppner, Elena Denisenko, Paul P Gardner, Sebastian Schmeier, Anthony M Poole

**Affiliations:** 1 Institute of Clinical Molecular Biology, Christian-Albrechts-University of Kiel, Kiel, Germany; 2 Institute of Natural and Mathematical Sciences, Massey University, Auckland, New Zealand; 3 Biomolecular Interaction Centre, School of Biological Sciences, University of Canterbury, Christchurch, New Zealand; 4 Bioinformatics Institute, School of Biological Sciences, University of Auckland, Auckland, New Zealand

**Keywords:** evolution, noncoding RNA, bioinformatics

## Abstract

Mammalian diversification has coincided with a rapid proliferation of various types of noncoding RNAs, including members of both snRNAs and snoRNAs. The significance of this expansion however remains obscure. While some ncRNA copy-number expansions have been linked to functionally tractable effects, such events may equally likely be neutral, perhaps as a result of random retrotransposition. Hindering progress in our understanding of such observations is the difficulty in establishing function for the diverse features that have been identified in our own genome. Projects such as ENCODE and FANTOM have revealed a hidden world of genomic expression patterns, as well as a host of other potential indicators of biological function. However, such projects have been criticized, particularly from practitioners in the field of molecular evolution, where many suspect these data provide limited insight into biological function. The molecular evolution community has largely taken a skeptical view, thus it is important to establish tests of function. We use a range of data, including data drawn from ENCODE and FANTOM, to examine the case for function for the recent copy number expansion in mammals of six evolutionarily ancient RNA families involved in splicing and rRNA maturation. We use several criteria to assess evidence for function: conservation of sequence and structure, genomic synteny, evidence for transposition, and evidence for species-specific expression. Applying these criteria, we find that only a minority of loci show strong evidence for function and that, for the majority, we cannot reject the null hypothesis of no function.

## Introduction

**Fig. 1.**
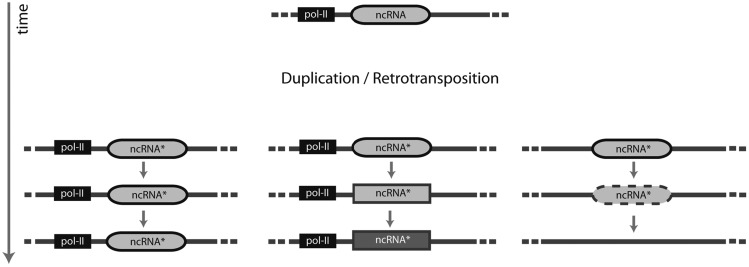
Duplicated RNA loci (ncRNA*) may follow one of several evolutionary trajectories. If expression is ensured through the presence of a promoter (e.g., pol-II), selection may act to maintain redundant loci if higher overall expression or expression of different loci under different conditions is beneficial (left). Alternatively, under relaxed or no selection, individual loci may start to diverge over time, and may in some cases take on new or altered biological roles (indicated here by rectangles or different shadings) which can again become subject to selection (middle). On the other hand, if expression cannot occur, a duplicated locus may be considered “dead on arrival” and is expected to decay (right).

With the initial sequencing of the human genome (Lander et al. 2001; Venter et al. 2001), it has become abundantly clear that only a very small fraction of the genomes of multicellular organisms is dedicated to making proteins; most genomes are largely comprised of various kinds of repetitive sequence, the majority of which possess an “organism-level” function (Palazzo and Gregory 2014). At the same time, it has become clear that there are numerous complex regulatory elements (Shlyueva et al. 2014) and noncoding RNAs (ncRNA; Ponting et al. 2009; Cech and Steitz 2014). NcRNAs have been shown to contribute to a range of integral cellular functions, including splicing (spliceosomal RNAs, snRNA), ribosome maturation (small nucleolar RNAs, snoRNA), and gene regulation (microRNA, miRNA; Cech and Steitz 2014). Interestingly, some of these families—most notably members of both snRNAs and snoRNAs—have undergone massive expansions during mammalian evolution (Marz et al. 2008; Schmitz et al. 2008; Hoeppner et al. 2009; Marz and Stadler 2009; Doucet et al. 2015), sometimes resulting in hundreds or thousands of unique loci per genome. The biological significance of this proliferation is however nontrivial to establish; it can be difficult to determine that a specific ncRNA locus contributes some function or—alternatively—is nonfunctional (fig. 1). Indeed, in some cases, expansions are best explained as being functionally neutral, with proliferation simply being the result of retrotransposition (Schmitz et al. 2008).

In contrast, some have argued, particularly for miRNA (Heimberg et al. 2008) and long-noncoding RNA (lncRNA; Mercer et al. 2009), that regulatory RNA diversification has been critical to increases in vertebrate complexity. Furthermore, maintenance of multiple copies of ncRNAs is in some cases known to be functionally important. For instance, following reduction of the ∼150 rDNA copies in *Saccharomyces cerevisiae* to half this number, the original copy number re-established (Kobayashi et al. 1998). While there is a requirement for production of ribosomes for protein synthesis, not all copies are transcriptionally active, yet reduced copy number strains show defects in damage repair, and the untranscribed copies appear to be critical for preventing premature separation of sister chromatids (Kobayashi 2011). A slightly less direct example of function is the SNORD116 snoRNA cluster associated with Prader-Willi syndrome in humans, which also appears to be developmentally important; deletion of the paternally inherited (but not the maternally inherited) snoRNA cluster results in postnatal growth retardation in a mouse model (Skryabin et al. 2007). It is however unclear whether it is one or more individual loci or a certain copy number that is required for function. In the case of SNORD116, this is complicated by evidence that this cluster is imprinted so copy number may be associated with conflict over parental-specific resource allocation, and may not exhibit novel function per se (Haig and Wharton 2003; Ubeda 2008). More generally, gene duplication may lead to the emergence of novel functions through neo-functionalization, boost expression levels, or give rise to more specialized functions through subfunctionalization (Ohno 1970; Lynch and Conery 2000). Importantly, the mode of proliferation, such as through transposon-dependent spread, should be considered as independent of function or lack thereof.

Several large-scale efforts have been undertaken in recent years to gather diverse data on a range of biochemical activities, including FANTOM (Forrest et al. 2014) and the ENCODE (Encyclopedia of DNA elements) project (EncodeProjectConsortium 2012), which aimed to identify all functional elements in the human genome. However, it has since become clear that a definition of function is not trivially derived from such data. A point of particular contention is the significance of individual biochemical signals versus the role of selection and conservation (Eddy 2013; Palazzo and Gregory 2014; Doolittle and Brunet 2017; Graur 2017). While the ENCODE project reported that any biochemical interaction may be interpreted as evidence for some level of function, an opposing view—held primarily within the field of molecular evolution—states that, in the absence of more direct functional tests, most of these signals may equally be explained as noise (see Doolittle 2013; Eddy 2013; Graur et al. 2013; Palazzo and Gregory 2014; Graur 2017 for discussion). However, most of the criticism has been at a conceptual level, and more detailed analyses of the data are warranted, given the disconnect between “biochemical evidence” and evolutionary conservation (Kellis et al. 2014). A critical insight from evolutionary theory is the adoption of a null hypothesis of no function, with rejection of the null hypothesis a critical step in assigning function (Gould and Lewontin 1979; Koonin 2016). Not being able to reject the null hypothesis does not demonstrate the absence of function for a given locus; further evidence may lead to rejection of the null and assignment of function. To make progress, it is thus critical to probe what we mean by function (Eddy 2013; Caballero et al. 2014; Doolittle et al. 2014; Graur et al. 2015), and to consider how to assess biological function in the age of “big data.” Indeed, “biochemical” data, such as evidence for expression, may be suggestive, but are alone not demonstrative of function, since such data may also result from biological noise.

In the spectrum of proposed functional elements, nontranslated transcriptional outputs such as ncRNAs represent a tractable starting point for developing tests of function. For the mammalian expansion of snRNAs and snoRNAs, given that both families fulfill their (canonical) biological role as transcribed molecules, functional copies should presumably show evidence of transcription as a minimal requirement for function. Not all transcriptional outputs are necessarily functional however (as the disconnect between the observation that ∼75% of the human genome is transcribed EncodeProjectConsortium 2012 and theoretical Graur 2017 and comparative genomic Lindblad-Toh et al. 2011 assessments indicating that <10% of the genome is under selection), so a clearer indication of function is conservation of expression. Nonfunctional copies may thus be expected to exhibit turnover across evolutionary time scales. With this in mind, we performed a comparative genomics analysis that integrated data from both ENCODE and FANTOM in an attempt to try to establish the evolutionary history and molecular signatures associated with function (if any) for a set of evolutionarily ancient, recently duplicated RNA genes. To ensure that our analyses are reproducible, we focused on highly standardized resources, taking biochemical data from ENCODE (EncodeProjectConsortium 2012) and FANTOM (Forrest et al. 2014) and genomic information from EnsEMBL (Yates et al. 2016).

Specifically, we examined five indicators: 1) positional conservation across multiple genomes, 2) evidence for independent expression, 3) evidence for conservation of expression, 4) evidence of transposon-mediated spread, and 5) how well individual ncRNAs fit curated reference (covariance) models in the RFam database (Griffiths-Jones et al. 2005; Nawrocki et al. 2015). We chose well-studied, well conserved and essential ncRNA families involved in splicing (snRNAs U1, U2, U4, U5, and U6) and ribosomal RNA maturation (snoRNA U3), as these represent core cellular functions, traceable to the Last Eukaryotic Common Ancestor (Davila Lopez et al. 2008; Marz and Stadler 2009; Hoeppner and Poole 2012) that have undergone recent copy number expansion in mammals. We find that, while some duplicated ncRNA loci, do show evidence consistent with function, these are in the minority, and we cannot reject the null hypothesis of no function for the majority of loci.

## Results

### Few Gene Loci Are Deeply Conserved

**Fig. 2.**
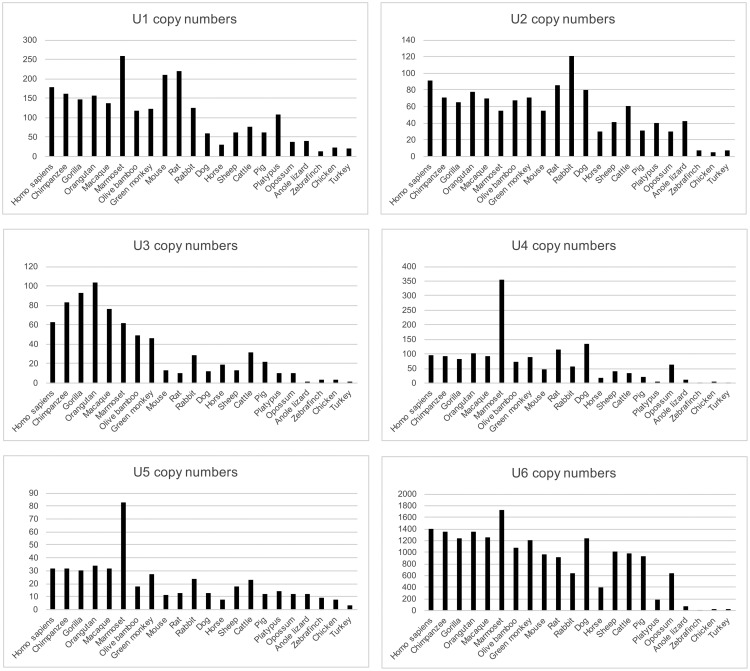
SnRNA and U3 snoRNA copy number variation across 23 amniote genomes (EnsEMBL release 83). U1–U3 small RNAs exhibit notable expansions with the advent of mammals, with individual families expanding to dozens (U1–U5) or hundreds (U6) of copies in any given genome.

**Fig. 3.**
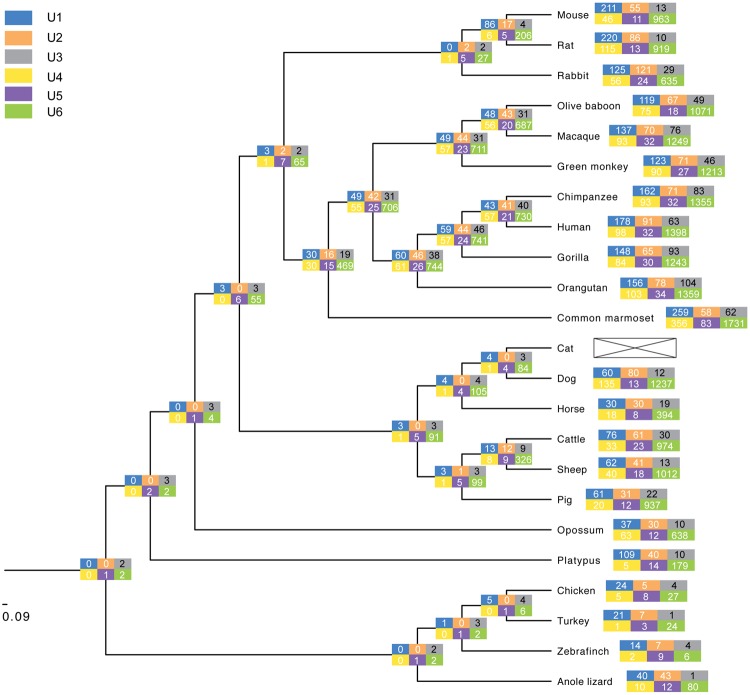
Estimating evolutionary conservation of individual ncRNAs using whole-genome alignments. Conservation of individual U1–U6 loci was reconstructed using a whole-genome alignment of 23 amniote genomes (EnsEMBL release 83). The RNA gene build for cat was absent from several releases in EnsEMBL, including the one used for this study, and is therefore not included in our reconstruction. Deep conservation to the amniote ancestor was tractable only for at most 1–2 copies per family, compatible with the notion of a recent expansion in the mammalian lineage and lack of long-term conservation of the resulting retrogene copies. Lack of deep conservation for some loci/families may be attributable to challenges in aligning a large number of genomes over comparably large evolutionary time scales.

Existing genome data and past analyses (Davila Lopez et al. 2008; Marz et al. 2008) show that U1 through U6 are present in multiple copies in the human genome (fig. 2). Of these, only a minority has been assigned an official name and status as functional gene by the HUGO Gene Nomenclature Committee (HGNC, http://www.genenames.org/; last accessed April 2017; supplementary figs. S2–S7, Supplementary Material online). One indicator of function is evolutionary conservation of a specific locus, suggesting the action of selection. If all loci were essential and performed distinct functions, this would be reflected in high levels of conservation of individual loci. We therefore performed synteny analysis across the 23 amniotes comparative genomic data set in EnsEMBL release 83 (Yates et al. 2016), spanning 19 mammals, 3 birds and the anole lizard. For the primate ancestor (50–55 Ma), ∼10% of human loci show positional conservation (fig. 3, supplementary table S1, Supplementary Material online). For the mammalian ancestor (∼200 Ma), this drops further and only between **0and3 loci** are conserved at this evolutionary depth—two orders of magnitude fewer than the numbers of loci in individual genomes (fig. 3).

A potential problem with comparative genome alignment data is that alignment quality depends on the degree of sequence conservation and may thus impact ancestral reconstruction of individual loci. To this end, we also performed pairwise alignments between human and mouse or chicken (EnsEMBL, data not shown). This gave much higher levels of conservation of loci. However, closer inspection revealed that the underlying algorithm (Harris 2007) actually aligns ncRNA loci from nonsyntenic regions (as judged from the flanking protein-coding genes)—an issue that can likely be attributed to difficulties stemming from the existence of dozens of highly similar loci across any two genomes. This approach thus provides a multitude of equally valid alignment options. In comparison, multi-species whole genome alignments need to reconcile a larger number of genomes, and use a different algorithm (Paten et al. 2008). Consequently, they are more strongly anchored by the more highly conserved protein-coding gene complement. A down-side of this approach is the loss of more divergent regions, measured as the overall whole-genome representation across species in the amniote data set (between 22% and 66% of any given genome, EnsEMBL FAQ). The multi-species amniote set thus provides a conservative estimate of deeply conserved loci, and is restricted to those loci that are readily traceable using standard comparative analyses.

To address whether the underlying alignment impacts our assessment of conservation, we next examined the evolutionary conservation of ncRNAs located within introns. Previous work indicates this subset of the data enables tracing of deep evolutionary conservation (Hoeppner et al. 2009; Hoeppner and Poole 2012), owing to the strong phylogenetic signal provided by the host genes. If our analysis is underestimating evolutionary conservation, we may expect to see a difference in the signal drop-off in the two data sets. However, among the few deeply traceable copies, we observe no clear pattern indicating that intronic loci are, per se, better conserved than intergenic loci (supplementary table S1, Supplementary Material online). Indeed, analysis of the most ancient loci detected in our synteny analysis indicates that some are in fact intergenic (supplementary tables S2–S13, Supplementary Material online).

### Autonomous Retrotransposons Play a Role in Copy Number Expansion of URNAs

The above analyses indicate that there are high copy numbers of each ncRNA family across mammals, yet high turnover of individual loci. However, it is unclear how high copy numbers are maintained. Individual redundant loci are not expected to be maintained by selection over evolutionary timescales (Nowak et al. 1997). Copy number increase in mammals may thus be a result of new loci being born at greater rates than they are lost. Alternatively, it may be that an individual locus is not important, but that copy number maintenance is important for function, as may to some degree be the case for rRNA (Ide et al. 2010). There are limited data on rDNA copy numbers (in fact, rDNA loci are often omitted from genome assemblies or represented by a single copy only, Zentner et al. 2011), but this can vary from <100 to >25,000 across plants and animals (Prokopowich et al. 2003). If amplification is critical for maintaining functional dosage, we might expect that the copy number of U3 snoRNA is similar to rDNA. For yeast versus human, this is not the case however. For all six ncRNAs under study, the copy number expansion appears to have occurred in the lineage leading to mammals (fig. 3).

The observed patterns of positional conservation above thus appear most compatible with a model of ongoing birth and death of individual RNA loci. We therefore sought to establish whether the ncRNA copies can be attributed to this. Looking at EnsEMBL ncRNA gene trees (Pignatelli et al. 2016), we find that the vast majority of RNA genes groups with homologs from one or several other species rather than within-species (data not shown due to complexity; trees are available for download at ftp://ftp.ensembl.org/pub/release-83/emf/ensembl-compara/homologies/). This finding is in line with the continuous emergence of individual loci along the branches of the mammalian phylogeny rather than evolutionarily recent bursts of copy numbers and their rapid decay.

It is well established that LINE element activity increase is associated with the emergence of the mammalian lineage (Waters et al. 2007). For ncRNA, copy number expansion can occur where a ncRNA is dispersed by the action of autonomous retrotransposons (Kordis et al. 2006; Schmitz et al. 2008; Doucet et al. 2015). This mode of integration generates distinct signatures of which the characteristic 3′ poly-A stretch is perhaps the bioinformatically most tractable (Jurka 1997; Esnault et al. 2000). (Other hallmarks, such as target site duplication, were found to be too variable in length and level of conservation for further analysis.) To gauge the level of LINE/L1 contribution to ncRNA mobility, we computed the fraction of adenosines in the 30 bp downstream flanking sequence of all U1–U6 loci. In line with our expectations, we see an adenosine excess (>50% of bases) for around 1/3 of all loci. Given that these signatures are comparatively short and are expected to decay rapidly in the absence of selection, this is likely to be an underrepresentation. As LINE activity has been associated with the emergence of Mammals (Richardson et al. 2015), copy number expansion appears to have been impacted by the activity of this class of retroelement, consistent with previous reports. Interestingly, in addition to LINE-mediated retrotransposition events, we also find a sizable fraction (∼20–40%, supplementary tables S2–S13, Supplementary Material online) of URNA loci to be directly flanked by repeat elements identified as LINE/L1 (supplementary fig. S1, Supplementary Material online). This suggests copies are hitch-hiking on the back of LINE retrotransposons. Alternatively, retrocopies may constitute hybrids/fusions between LINE/L1 and URNA transcripts due to template switching, as has previously been reported (Garcia-Perez et al. 2007).

### Most ncRNA Loci Show No Evidence of Independent Transcription

LINE/L1 expression may impact the genomic copy number of URNAs, but is agnostic with regard to function of individual loci. However, given that retrotransposition is expected to disconnect a displaced copy from its regulatory context and associated promoter, we speculate that many retrotransposed small RNA genes could be “DOA” (dead on arrival)—consistent with high copy number turn-over. To assess whether individual copies are “DOA,” we examined evidence for locus expression. We did this in two complementary ways. Some ncRNA, such as U1–U5 (Hernandez 2001; Egloff et al. 2008) but not U6 (Brow and Guthrie 1990), are known from previous work to be expressed in a Pol-II-dependent manner, so we used ENCODE Pol-II ChIPseq data from seven human cell lines, and five mouse cell lines (EncodeProjectConsortium 2012; Landt et al. 2012) to assess whether individual copies are associated with annotated Pol-II promoters. We also used a number of transcriptome data sets for both mouse and human to independently assess locus expression (see Materials and Methods).

Across all six families, the majority of loci has no ENCODE-annotated pol-II sites within 500 bp from the transcription start site (TSS; table 1). For cases where there was evidence of pol-II binding in only a single cell line, the proportions of loci spanned from under 10% to around 40%. If the criterion that pol II-binding evidence should span all cell lines is included the numbers drop to below 25%. As a control, our data for U6 snRNA (which is not expected to exhibit pol II-dependent expression) show that only 2/1,397 copies in human and 0/964 copies in mouse show broad support for a colocated pol-II binding-site. Taken together, this suggests that only a minority of loci have the potential for pol-II-dependent expression. It is of course also possible that that pol-II binding for a subset of these loci is restricted to cell-lines and/or conditions not probed by the ENCODE project.

**Table 1.** Fraction of Loci in Human or Mouse with Putative Pol-II Promoter Element within 500 bp of Transcription Start Site.

	Human			Mouse		
	No Pol-II Site (%)	At Least One Cell Line (%)	All Cell Lines (%)	No Pol-II Site (%)	At Least One Cell Line(%)	All Cell Lines (%)
U1	127 (71)	51 (29)	30 (17)	173 (82)	38 (18)	12 (6)
U2	54 (59)	37 (41)	15 (16)	36 (66)	19 (35)	1 (1.8)
U3	53 (84)	10 (16)	7 (11)	4 (31)	9 (69)	4 (31)
U4	88 (91)	9 (9)	2 (2)	42 (91)	4 (8)	2 (4)
U5	23 (72)	9 (28)	8 (25)	4 (36)	7 (64)	1 (9)
U6	1355 (97)	42 (3)	2 (0.1)	923 (96)	40 (4)	0 (0)

However, expression may occur via other routes, such as splicing-dependent expression for the ∼50% of loci located in the introns of other genes (supplementary tables S1–S12, Supplementary Material online; Runte et al. 2001; Rodriguez et al. 2004; Yin et al. 2012), from pol-III, or from more distant promoter elements. We therefore examined whether other experimental data could confirm expression for individual loci. To this end, we used 45 and 128 RNA-seq data sets from the ENCODE project as well as 931 and 966 publicly-available CAGE data sets for mouse and human, respectively (Forrest et al. 2014). In combination, these types of data should in principle capture all expression, regardless of promoter type. That said, because expression from short read data is determined by the number of reads mapping to individual loci, we wondered if the frequent duplication of small RNAs may impact our ability to accurately detect locus-specific transcription signals; owing to sequence similarity between loci, we might expect that some proportion of reads map ambiguously (i.e. to multiple loci). We were thus first interested in determining the sequence diversity within a given family to gauge the possibly of unambiguously assigning reads to unique loci. To this end, we generated sequence alignments for each family and calculated the pairwise number of nucleotide differences for any two sequences. As summarized in table 2, mean pairwise distance varies across and within families, ranging from 50.53 (±12.98) differences for U5 up to 100.97 (±41.36) for U2 snoRNAs.

**Table 2.** Mean Number of Pairwise Differences and Mean SNP Density for Loci of Human and Mouse URNA Families.

	Mean No. of Pairwise Differences (SD)		Mean # SNPs (SD)	
	Human	Mouse	Human	Mouse
U1	74.78 (25.41)	85.44 (20.95)	3.7 (4.6)	5.1 (3.6)
U2	100.97 (41.36)	105.35 (35.62)	4.8 (9.4)	4.8 (3.0)
U3	79.21 (16.74)	45.57 (37.40)	9.4 (12.1)	5.5 (5.1)
U4	83.74 (32.39)	101.02 (23.30)	5.0 (8.3)	5.5 (3.5)
U5	52.53 (12.98)	30.10 (12.31)	14.5 (22.5)	4.1 (3.1)
U6	53.92 (13.71)	54.02 (17.98)	3.0 (2.5)	3.3 (2.6)

**Fig. 4.**
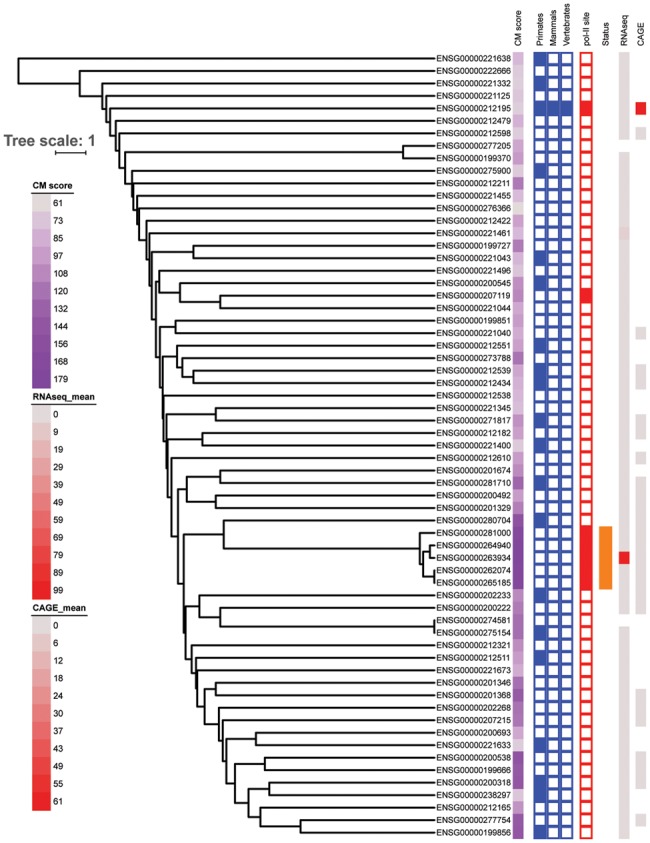
Tree of pairwise similarities of human U3 snoRNA copies and their expression across multiple samples combined with annotation (CM) score, depth of conservation across 23 amniote genomes (columns 2–4), presence of pol-II sites within 500 bp of the transcription start in at least 5 out of 7 probed cell lines, status in HGNC (solid = known, missing = not listed in HGNC) and mean expression from RNA-seq and CAGE data (see Materials and Methods for details). Missing expression estimates correspond to genes not located on the primary assembly or genes without unambiguous expression estimates.

For RNA-seq data, using tools and settings established by the ENCODE project (see Materials and Methods) in combination with a conservative (which we deem necessary given the potential issues arising from multi-mapped reads) tool for translating read alignments to expression estimates (Roberts et al. 2011; Anders et al. 2015), we find that only a subset of loci show signals suggestive of transcription (supplementary File S1, Supplementary Material online), although the specific picture differs somewhat depending on the RNA family, ranging from only a few putatively expressed loci (i.e. human U3 snoRNAs, fig. 4) to somewhat diffuse signals covering multiple loci at comparable expression levels (i.e. U1; supplementary File S1, Supplementary Material online). CAGE data paint a very similar picture when using an equally stringent counting approach, with only a subset of loci showing some indication of expression. CAGE and RNA-seq results are not fully congruent, which can likely be attributed to differences in the underlying technical approach (full gene mapping in RNA-seq versus short 5′ tags in CAGE) and/or actual biological differences in the various underlying (largely nonoverlapping) samples (supplementary figs. S2–S13, Supplementary Material online).

**Fig. 5.**
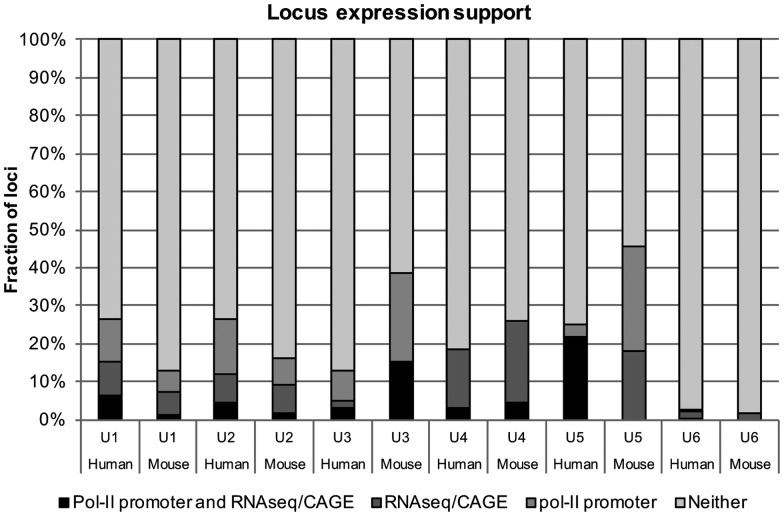
Evidence for locus transcription. We used two independent measures to determine potential expression of annotated URNA loci in human and mouse—the presence of a predicted upstream pol-II promoter element (≤500 bp) and signals from RNA-seq data and/or CAGE (see Materials and Methods). The data suggest that a minor fraction of loci for each family has support from both lines of evidence (“Pol-II Promoter and RNA-seq/CAGE”), whereas a sizable number at least have a putative promoter (“pol-II promoter”) suggesting that a locus may be transcriptionally active but perhaps not under the observed conditions. Another 5–15% of loci have mapped reads (“RNA-seq/CAGE”), but no nearby promoter candidate, which may hint at either another means for transcription or stochastic effects of read mapping against highly similar gene copies. Lastly, the majority of loci across families has no support for transcription whatsoever, strongly indicating that they are inactive retrogenes.

We next wanted to test whether these two methods for gauging transcriptional activity (i.e. pol-II promoter mapping and sequencing-based expression assays) correlate. Figure 5 shows that, for most families, <10% of loci show expression in RNA-seq/CAGE data while also having an adjacent putative pol-II promoter. In turn, a sizable fraction of loci has at least one pol-II promoter candidate without strong evidence for expression through transcriptomics data. This finding could suggest that expression is perhaps restricted to cell types not considered in our sample of RNA-seq and CAGE data or else that the presence of a promoter element alone is not in itself an unambiguous indicator of activity. Overall however, these analyses indicate that the majority (ranging from ∼60% for human U3 to ∼95% for human and mouse U6) of loci in both human and mouse does not show evidence of expression from any of the available data (supplementary tables S2–S13, figs. S2–S13, Supplementary Material online), meaning we cannot reject the null hypothesis of no function for these loci. When focusing on those URNA loci that have been classified as functional through independent annotation efforts (HGNC, MGI—Mouse Genome Informatics, http://www.informatics.jax.org/; last accessed April 2017), we find that out of the 34 “known” human URNAs (U1: 16 loci, U2: 1 locus, U3: 5 loci, U4: 2 loci, U5: 5 loci, U6: 5 loci), the majority (30/34) has strong support for the presence of an associated pol-II promoter and score (with some notable exceptions) within the top 5% of each families’ respective highest annotation score using so-called covariance-models (CM, yielding a score that describes goodness-of-fit to the reference alignment/structure on which the model is based). Expression, however, was only detectable for 15/34 loci (RNA-seq: 12, CAGE: 8) using our stringent mapping approach. Likewise, only 8 out of 34 functional candidates show conservation across mammals whereas the rest appears species-specific on the basis of the 23 amniote whole genome alignment.

### Sequence Conservation Can Illuminate Evolutionary Trajectories for Redundant Small RNA Genes

A standard way to identify RNA gene homologs is through similarity searches, an approach that underpins the annotation of RNA genes in genomes (Griffiths-Jones et al. 2005; Nawrocki et al. 2015). However, as RNA genes lack open reading frames, it can be nontrivial to distinguish functional copies from nonfunctional pseudogenes or from divergent RNAs with distinct functions. This caveat notwithstanding, it is possible to assign scores to predicted RNA gene loci, based on how well they match a corresponding, manually curated covariance model (CM), accounting for both primary and secondary sequence features (Griffiths-Jones et al. 2005; Nawrocki et al. 2009).

**Fig. 6.**
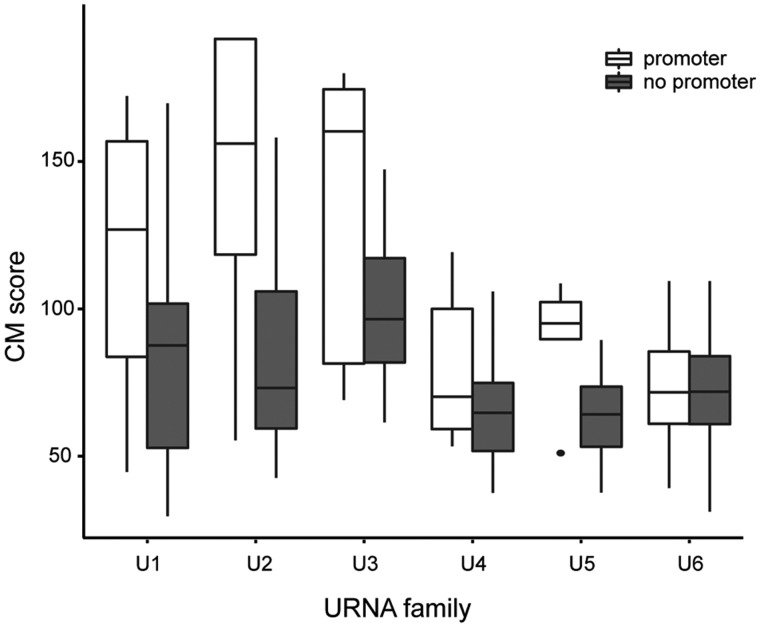
Rfam (release 12.1) CM scores for human U1–U6 loci (EnsEMBL version 83) with and without predicted upstream pol-II promoter. A comparison of annotation scores (higher = better) for six families of frequently duplicated small RNAs shows good correlation with promoter presence as indicator for function in U1, U2, U3, and U5 (Kolmogorov–Smirnov, *P* < 0.05) and no clear correlation for snRNAs U4 and U6 (Kolmogorov–Smirrnov, *P* > 0.5), the latter of which is known to not be transcribed by pol-II but pol-III. This finding suggests that promoter presence can be an important factor in identifying functional from nonfunctional (and thereby likely decaying) copies.

To assess whether pol-II-associated ncRNAs are more likely to be functional than those with no association to observed pol-II promoters, we ranked each locus against a CM of verified reference genes (Nawrocki et al. 2015). CM scores should provide an indication of possible divergence from the known (reference) function. We split the loci into those for which there was evidence of pol-II activity, and those for which there was none, and we plotted CM scores. With the exception of U6, which is not known to be expressed by pol-II and should therefore not show any correlation, and U4, the distributions of CM scores for expressed loci were significantly higher than those lacking evidence of pol II-associated activity (fig. 6; Kolmogorov–Smirnov: *P* < 0.05).

To examine whether there is evidence for selection or decay, we next analyzed the patterns of sequence variation across human ncRNA loci using the 1,000 human genomes reference data set (Genomes Project et al. 2015). Under our narrow definition, copies under relaxed selection are expected to decay through accumulation of mutations over time until they are no longer recognizable. Evidence of functional constraint (purifying selection) may be manifested through a marked difference in the amount of observed variation in deeply conserved loci (no or low variation) as opposed to very young loci (high variation). In contrast, comparable levels across loci could suggest a degree of robustness of these ncRNAs to random nucleotide changes (given ∼25% of changes in ncRNAs are functionally neutral, Kun et al. 2005) or that most loci are in fact subjected to the same rate of mutation, compatible with them having no selected function.

To distinguish between these cases, we only examined loci that are present in human plus at least one other primate in our comparative genomics analysis of 23 amniotes. We reasoned that longer-lived loci may be less likely to be undergoing lineage-specific functional diversification. We find that for the URNAs used in this study, the average number of variants per gene locus varies between 3.0 for U6 and 14.5 for U5 (table 1, supplementary fig. S8, Supplementary Material online), similar to estimates obtained for mouse (table 1). We also observe a few very significant deviations, particularly for an ancient U3 locus (ENSG00000212195), where data from phase 3 of the 1,000 genomes project (Genomes Project et al. 2015) suggest the presence of 78 small variants (supplementary tables S2–S7, Supplementary Material online) with a minor allele frequency over 0 in at least one of the five studied populations (AFR, AMR, EAS, EUR, and SAS). This particular finding could suggest relaxed selective constraint on the locus, as would be expected for a decaying retrogene. However, since the majority of other loci shows much lower rates of variation, one may speculate that the seemingly increased variant load for the TEX14-associated copy is potentially compatible with sub or neofunctionalization. Short of designing a functional assay to verify “U3” functionality, this point remains speculative, however.

## Discussion

### The Majority of URNA Copies Is Likely Not Functional

We have examined a range of evidence from highly standardized consortia data sets (ENCODE and FANTOM) for Mouse and Human, homology search tools and comparative genome alignments to assess function of individual ncRNA loci across mammals. Interestingly, we see clear correlation between the presence of an annotated pol-II promoter in several URNA families and RNA-seq/CAGE signals indicative of expression but also with high annotation (CM) scores. On this criterion, there is insufficient evidence for between 60% and 95% of all URNA loci to reject the null hypothesis of no function. While the criteria we use (expression, synteny) span multiple forms of evidence, this does not preclude the possibility that additional data might increase the number of functional copies. For example, counting only uniquely mapping short reads (see Materials and Methods) may underestimate expression for highly similar loci. Longer sequencing reads may mitigate this problem to some extent, as a greater proportion of reads may be uniquely mapped. That said, employing additional criteria for function may be needed. Indeed, some authors have gone to impressive lengths to assess function. Lewejohann et al. (2004) demonstrated function using a battery of behavioral tests for a particularly recalcitrant ncRNA, BC1 in mice. We note that this ncRNA was nevertheless expressed, so would be captured by our informatics-based approach.

Therefore, while our data lend support to the view that retroposed ncRNA genes are “dead on arrival,” our findings also suggest that there is some level of evidence for multiple functional copies per URNA family. Several loci across the six URNA families studied here are presumably functional on the basis of independent curation efforts (HGNC, MGI) and also exhibit very high CM scores, but do not meet (some of) our key informatics criteria for function—most notably expression and/or deep conservation (supplementary tables S2–S13, figs. S2–S13, Supplementary Material online). We can see several plausible explanations for this result. First, while sequence divergence overall is high within each RNA family, putatively functional loci often have one or more near-identical paralog. This finding is expected, as these loci—given that they are actively expressed,—are the most likely source of new paralogs. However, this could mask signals derived from RNA-seq and/or CAGE analysis under our very stringent mapping rules (required to ensure unique mapping and to eliminate multi-mapping ambiguities). Secondly, the majority of known functional loci in human and mouse is intergenic and located in unaligned regions across our 23 amniote data set, thus appearing as species-specific rather than deeply conserved. Thirdly, expression of loci may be tissue-specific and not effectively captured by the data sets compiled for the FANTOM and ENCODE projects. No doubt, future efforts will help shed further light on this issue as more well-integrated data become available. Finally, there is also a small chance that some of the known HGNC loci constitute very recently retrotransposed pseudogenes and were erroneously annotated as functional based on sequence-analysis alone.

Clearly, boundaries between functional and nonfunctional loci appear fluid on the basis of the various lines of evidence used in our study. While data derived from large-scale studies, specifically ENCODE and/or FANTOM, allow us to draw a rich map of signals related to function and provide valuable guidance towards assigning functional status to the various transcribed elements in a genome, our results also highlight potential pitfalls and limitations when trying to distinguish functional genes from paralogous, nonfunctional retrogenes on the basis of computational analyses alone.

### Some Paralogs May Be Candidates for Neofunctionalization

While the majority of loci for which expression could be established (see above) also score highly against the respective CM profile, our analyses do reveal some instances of paralogs that score poorly against their respective CMs, but which also show deep conservation and some evidence of expression (supplementary tables S2–S13, Supplementary Material online). On the basis of our classification for the evolutionary trajectory of redundant URNAs, these may thus represent cases of functional divergence.

**Fig. 7.**
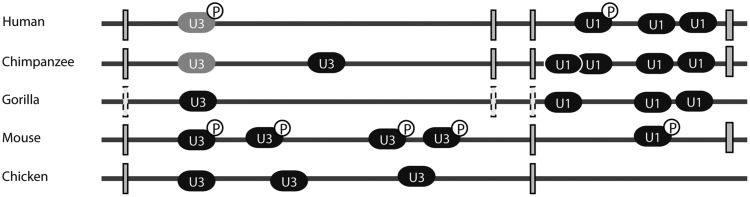
The testis-expressed gene 14 (TEX14) exhibits a remarkable relationship with ncRNA genes over the course of vertebrate evolution, hosting varying numbers of both U1 snRNA and U3 snoRNA copies across different species. Data from the ENCODE project for mouse and human suggest that not all loci are necessarily functional, lacking evidence for the presence of an associated pol-II promoter (P). Interestingly, U3 copies in both human and chimpanzee share a large deletion (grey) while still being expressed. It is not currently possible to determine whether this is a prelude to loss of the copy or an indication of the emergence of novel function. Perhaps arguing for the latter, the TEX14 gene in gorilla has been shown to carry a loss-of-function mutation (dashed outline), whereas the embedded U3 snoRNA remains intact, essentially turning the locus “inside-out.”

One particularly intriguing example is the U3 locus (ENSG00000212195) in the second intron of TEX14 (fig. 7), a testis-expressed gene encoding a kinase that is conserved across terrestrial vertebrates (EnsEMBL release 83). Interestingly, this is the oldest U3 locus in our comparative analysis (supplementary tables S2–S13, Supplementary Material online), yet it received a low CM score (CM score: 69), indicating a poor fit to the U3 family. Given that this U3-like sequence is conserved across amniotes, and data from both Mouse and Human support pol II-dependent expression, the TEX14 locus might be a good candidate for neofunctionalization in the presence of additional, higher scoring loci with even stronger support for expression (e.g., ENSG00000265185, CM score of 174) and which we expect to be equally ancient, but which are located in an unaligned region of the genome. Closer inspection reveals the score-diminishing variation to be a large deletion of a stem-loop structure outside of the key C/D box motif (supplementary fig. S15, Supplementary Material online). Interestingly, this deletion is also present in chimpanzee, suggesting the origin of the deletion to be in the common ancestor of these species. Given the otherwise strong conservation of the whole gene sequence and its predicted secondary structure, it is unclear whether the deletion significantly alters the function of the U3 copy or indeed renders it a pseudogene. The SNP load for this particular locus in human is clearly elevated (78 SNPS in g1k), and inspection of mapped reads shows mapping for this locus to be generally unique, likely as a result of the strongly distinguishing deletion. As such, it is likely that the locus is indeed in the process of being lost due to relaxed selection.

It is intriguing to note that in Gorilla, the TEX14 gene has pseudogenized (Scally et al. 2012), but the U3 gene appears intact. TEX14 furthermore appears to be a popular location for URNAs, with three copies of U1 present in the first intron of human (fig. 7). Depending on the organism, we see multiple copies of both U1 and U3 genes in TEX14 introns. We speculate that this may be a result of germline expression as TEX14 is exclusively and highly expressed in testis (41 FPKM in ENCODE). Consequently, this gene may be an ideal target for heritable retroinsertion of these highly expressed ncRNAs. However, we also note that a cursory analysis of other germ-line expressed genes did not show this to be a consistent pattern but one that appears linked to TEX14 specifically. The variation we see in copy number in some species may therefore be an effect of transcriptional proximity and expression level.

## Concluding Remarks

In this paper, we have examined the proliferation of ncRNA copy number as a means to help advance the question of how to assign (or discount) function to noncoding elements. We approached the issue by combining tools from both high-throughput data analysis and evolutionary analysis. Our findings reveal a complex landscape of evidence from both genomic expression data and comparative genomic analyses. Whereas few of the copies appear functional on the analyses performed here, no single line of evidence could be identified that provided unambiguous signals to classify a locus as functional or nonfunctional. That said, the incorporation of comparative data, in the form of genome-scale alignments and CM, combined with expression data generated via ENCODE and FANTOM, improves our capacity to identify functional candidates. While it may be the case that many loci lack the strong signals consistent with function, these may still turn out to have some function. Integration of additional expression data sets that allow an even finer resolution of the spatial and temporal patterns of gene activity may further increase the number of functional candidates. However, as noted above, the caveat here is that expression data alone do not provide unambiguous evidence for function—detailed experimental assessments of function for species-specific loci are necessary. At the same time, expanding the efforts of ENCODE and FANTOM to include additional (vertebrate) model systems will help in identifying both patterns of conservation and expression, and may increase confidence in the functional status of individual loci. Regardless of the data type, it is critical to frame the assignment of function in the context of a null hypothesis: for the majority of loci examined here, and using the tests we employed, we were not able to reject the null hypothesis of no function.

## Materials and Methods

### Annotation of ncRNA Genes

Annotations for U1–U6 sn(o)RNAs were retrieved from the EnsEMBL database using the public Perl API (release 83). The EnsEMBL ncRNA annotation pipeline relies on both publicly available gene models (i.e. HGNC) as well as on the prediction of candidate gene structures using manually curated and thresholded CM from the RNA family database RFam (http://dec2015.archive.ensembl.org/info/genome/genebuild/ncrna.html; last accessed April 2017). For the expression analysis (see below), boundaries for EnsEMBL ncRNA models were recomputed using the Infernal package and covariance models from RFam release 12.1 (see below) to correct minor issues with start/stop coordinates in a small number of loci annotated in EnsEMBL.

### Synteny Analysis

Synteny was established based on the multi-genome “23 amniotes” PECAN alignment available through the EnsEMBL Compara database release 83 (Yates et al. 2016). Briefly, we iterated over all species in the data set, querying all ncRNA genes belonging to a given RFam family. For each gene locus, we retrieved all positionally overlapping gene models from the other 22 species to construct syntenic groups. Each gene recovered in this way was then removed from the search space to prevent subsequent, reciprocal hits, until all ncRNA genes had been assigned to a group or remained as singletons.

### Ancestral State Reconstruction of Aligned ncRNA Loci

Individual syntenic groups were translated into a presence–absence matrix and used to perform ancestral state reconstruction with dollo parsimony from the Phylip package (Felsenstein 2009). Dollo specifically excludes any prior assumption about the gain and loss of loci, an approach which we deem sensible given that, to the best of our knowledge, no such model exists to accurately describe the dynamics of retrotransposing ncRNA.

### CM Scores

Annotation scores for existing annotations in the EnsEMBL database were computed using Infernal (v1.1) based on the respective RFam covariance model (RFam version 12.1) against the EnsEMBL gene model plus 100 bp of flanking sequence to avoid truncating the predicted gene models.

### RNA Alignments and Phylogenetic Trees

RNA sequences, based on our customized annotations, where aligned using Muscle (Edgar 2004) to determine pairwise distances and compute trees for visualization based on average sequence similarity in percent (Waterhouse et al. 2009).

### Repeat Annotations

We searched for repeat features in the 100 bp flanking regions of EnsEMBL ncRNA gene models using RepeatMasker (version 4.0.3) against the human repeat database distributed through grinst.org (release 2016 Aug 29).

### Expression of Small RNAs Using RNA-Seq

Expression of U1–U6 was determined using all samples from the human ENCODE smallRNA-seq data set (tissues only) and a subset of mouse ENCODE totalRNA libraries (supplementary tables S13 and S14, Supplementary Material online). Reads were processed using tools and settings established and published by the ENCODE project against the human genome assembly GRCh38 and the mouse genome assembly GRCm38, respectively (EnsEMBL release 83).

Considering that U1–U6 occur in numerous copies, we elected to only count reads that could be uniquely mapped. This is in contrast to defaults used by the ENCODE projects where individual reads may map to up to 20 positions as long as other thresholds with regards to base-pair mismatches are obeyed. Here, expression was instead quantified using the HTSeq package in combination with our updated URNA annotations (see above), which returns the number of reads aligning to a given locus while rejecting all reads with more than one equally valid mapping location. From these counts, we derived RPKM values using the formula [reads_at_locus/(number_of_mapped_reads/1000000)]/length_of_gene_in_kb. While this stringent approach is likely to underestimate expression for recently duplicated but potentially functional copies, competing methods that allow multi-mapped reads are expected to report expression for copies even if these are not actually functional (tested with Cufflinks version 2.2.1; data not shown). Here, we elected to use the conservative approach, favoring false negatives over false positives.

### Expression of Small RNAs Using CAGE

Mouse genomic coordinates (mm10) and tag counts of cap analysis of gene expression (CAGE) TSSs were obtained from the FANTOM5 project (Forrest et al. 2014) data repository (http://fantom.gsc.riken.jp/5/datafiles/reprocessed/mm10_v2/basic/; last accessed June 2017). The DPI beta program (https://github.com/hkawaji/dpi1/; last accessed June 2017) was used as described in Forrest et al. (2014) to cluster mouse CAGE TSSs into CAGE peaks. For human, CAGE data mapped to hg19 were downloaded from http://fantom.gsc.riken.jp/5/datafiles/phase1.3/basic/; last accessed June 2017. Permissive CAGE peaks were downloaded from http://fantom.gsc.riken.jp/5/datafiles/phase1.3/extra/CAGE_peaks/; last accessed June 2017. Genomic coordinates were converted from hg19 to hg38 using the liftOver program (https://genome.ucsc.edu/cgi-bin/hgLiftOver; last accessed June 2017). For sample names/accession numbers, please see supplementary tables S15 and S16, Supplementary Material online.

We excluded CAGE peaks located on the same strand within 500 bp of start sites of protein-coding transcripts (EnsEMBL release 83). We next assigned a CAGE peak to a snRNA if their 5′ ends were located within 500 bp on the same strand. Tag counts of CAGE peaks associated to the same snRNA were summed up. CAGE peaks that could not be uniquely assigned to a single snRNA, samples with expression in less than two snRNAs, and snRNAs with expression in less than two samples were excluded. Data were normalized to tags per million (TPM) using TMM normalization procedure (Robinson and Oshlack 2010).

## Acknowledgments

AMP acknowledges support of the Royal Society of New Zealand from a Rutherford Discovery Fellowship (RDF-11-UOC-013).

## Supplementary Material

Supplementary data are available at *Molecular Biology and Evolution* online.

## Supplementary Material

Supplementary Data
