# Supplementary material for: An Evaluation of Function of Multicopy Noncoding RNAs in Mammals Using ENCODE/FANTOM Data and Comparative Genomics
Source: Mol Biol Evol. 2018 Apr 3;35(6):1451–62. doi: 10.1093/molbev/msy046 (PMC5967550; doi:10.1093/molbev/msy046)
Supplement: Supplementary Data [file msy046_supp.zip › Hoeppner_etal_MBE_Supplementary.pdf]

An evaluation of function of multicopy non-coding RNAs in mammals using ENCODE/FANTOM data and comparative genomics.

Marc P. Hoeppner[1]*, Elena Denisenko[2], Paul P. Gardner[3], Sebastian Schmeier[2], Anthony M. Poole[4]*

[1]Institute of Clinical Molecular Biology, Christian-Albrechts-University of Kiel, Kiel, Germany
[2]Institute of Natural and Mathematical Sciences, Massey University, Auckland, New Zealand
[3]Biomolecular Interaction Centre, School of Biological Sciences, University of Canterbury, Christchurch, New Zealand
[4]Bioinformatics Institute, School of Biological Sciences, University of Auckland, Auckland, New Zealand

*Correspondence: m.hoeppner@ikmb.uni-kiel.de or a.poole@auckland.ac.nz

Supplementary information

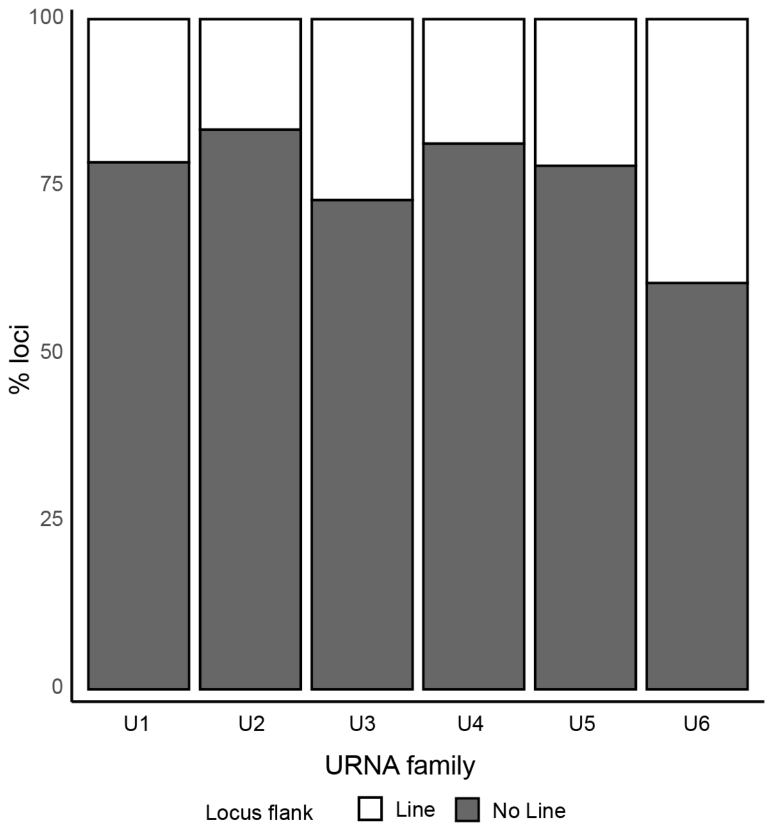

Figure S1: Fraction of human ncRNA loci with flanking LINE/L1 features. The activity of retrotransposons such as LINE/L1s has been associated with the proliferation of non-coding RNA genes in the mammalian lineage. Correlating ncRNA annotations with adjacent L1-like repeat sequences suggests that up to 40% of URNA annotations are associated with L1 features, in line with L1 mediated mobility.

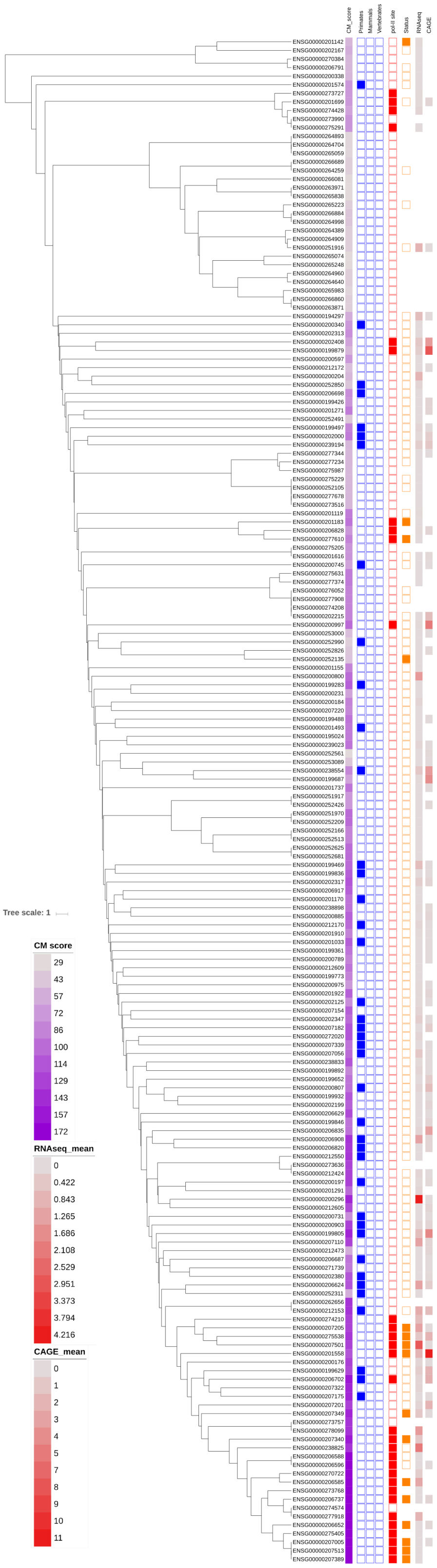

Figure S2: Tree of pairwise similarities of human U1 snRNA copies with (from left to right): 1) CM score (purple), conservation of locus in 2) primates, 3) mammals or 4) vertebrates, 5) presence of pol-II promoter upstream of locus, 6) status of gene locus in HGNC (solid = known, empty = known pseudogene, missing = not listed in HGNC) and mean expression from 7) ENCODE small RNAseq and 8) CAGE data (see Methods). Missing expression data = gene not on primary assembly or no unambiguous call was possible to due redundant mappings.

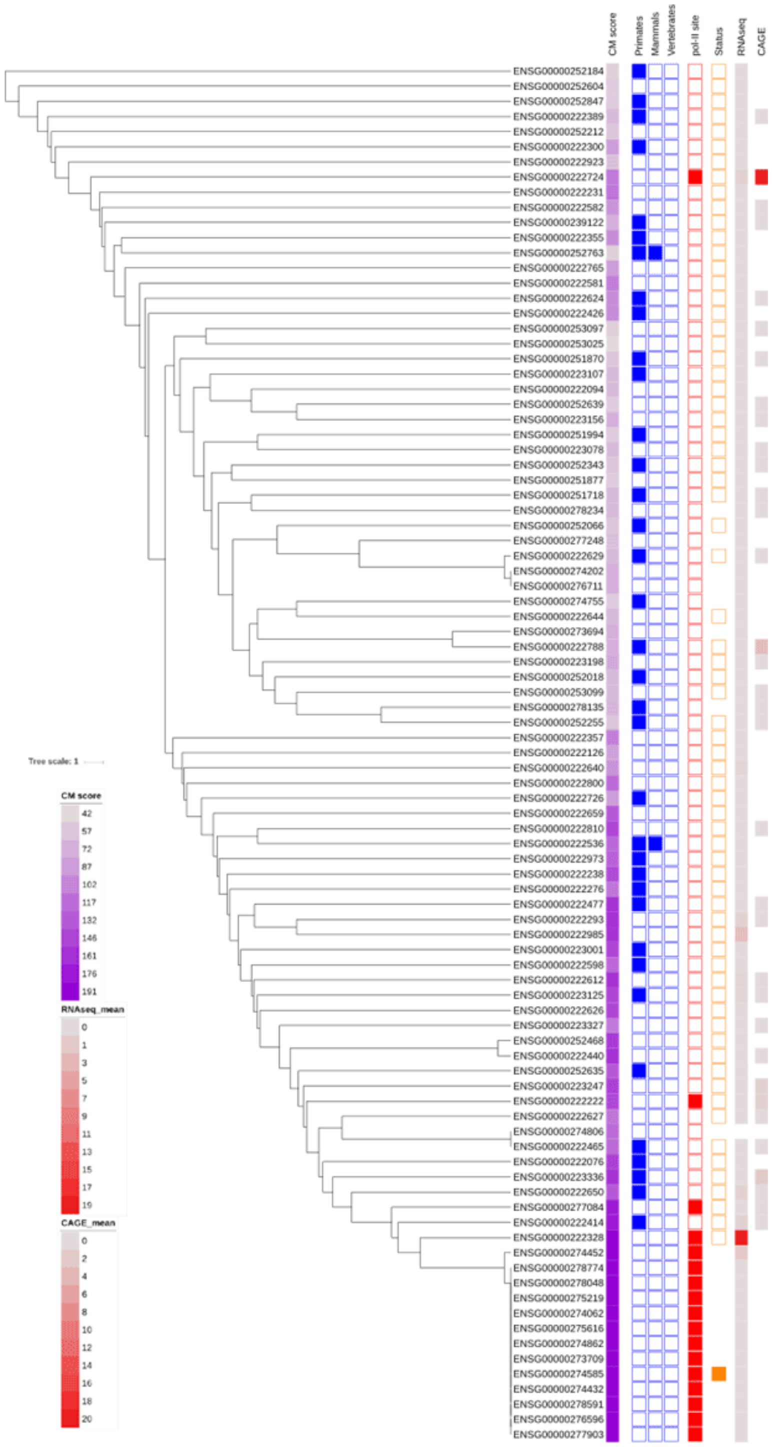

Figure S3: Tree of pairwise similarities of human U2 snRNA with (from left to right): 1) CM score (purple), conservation of locus in 2) primates, 3) mammals or 4) vertebrates, 5) presence of pol-II promoter upstream of locus, 6) status of gene locus in HGNC (solid = known, empty = known pseudogene, missing = not listed in HGNC) and mean expression from 7) ENCODE small RNAseq and 8) CAGE data (see Methods). Missing expression data = gene not on primary assembly or no unambiguous call was possible to due redundant mappings.

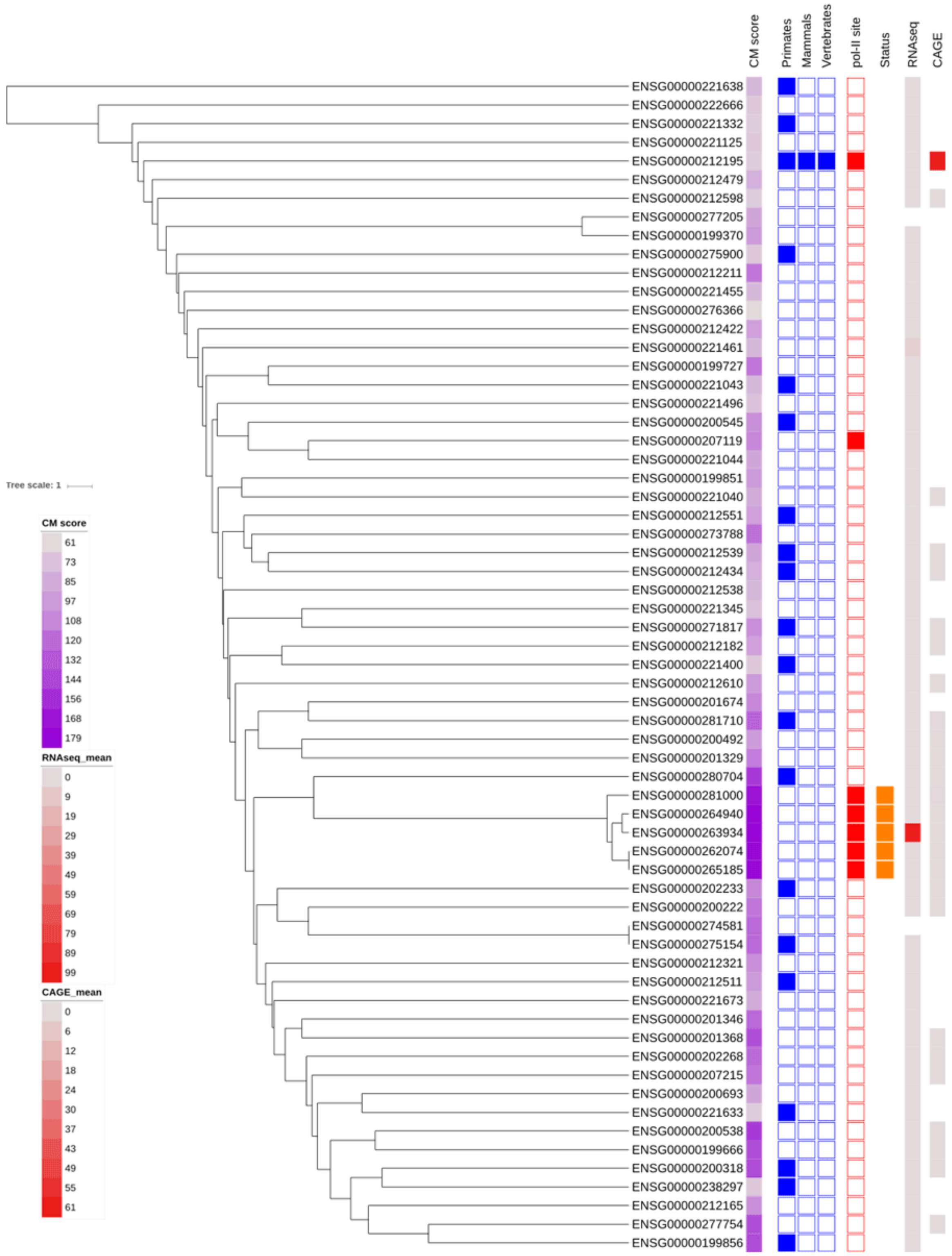

Figure S4: Tree of pairwise similarities of human U3 snoRNA copies with (from left to right): 1) CM score (purple), conservation of locus in 2) primates, 3) mammals or 4) vertebrates, 5) presence of pol-II promoter upstream of locus, 6) status of gene locus in HGNC (solid = known, empty = known pseudogene, missing = not listed in HGNC) and mean expression from 7) ENCODE small RNAseq and 8) CAGE data (see Methods). Missing expression data = gene not on primary assembly or no unambiguous call was possible to due redundant mappings.

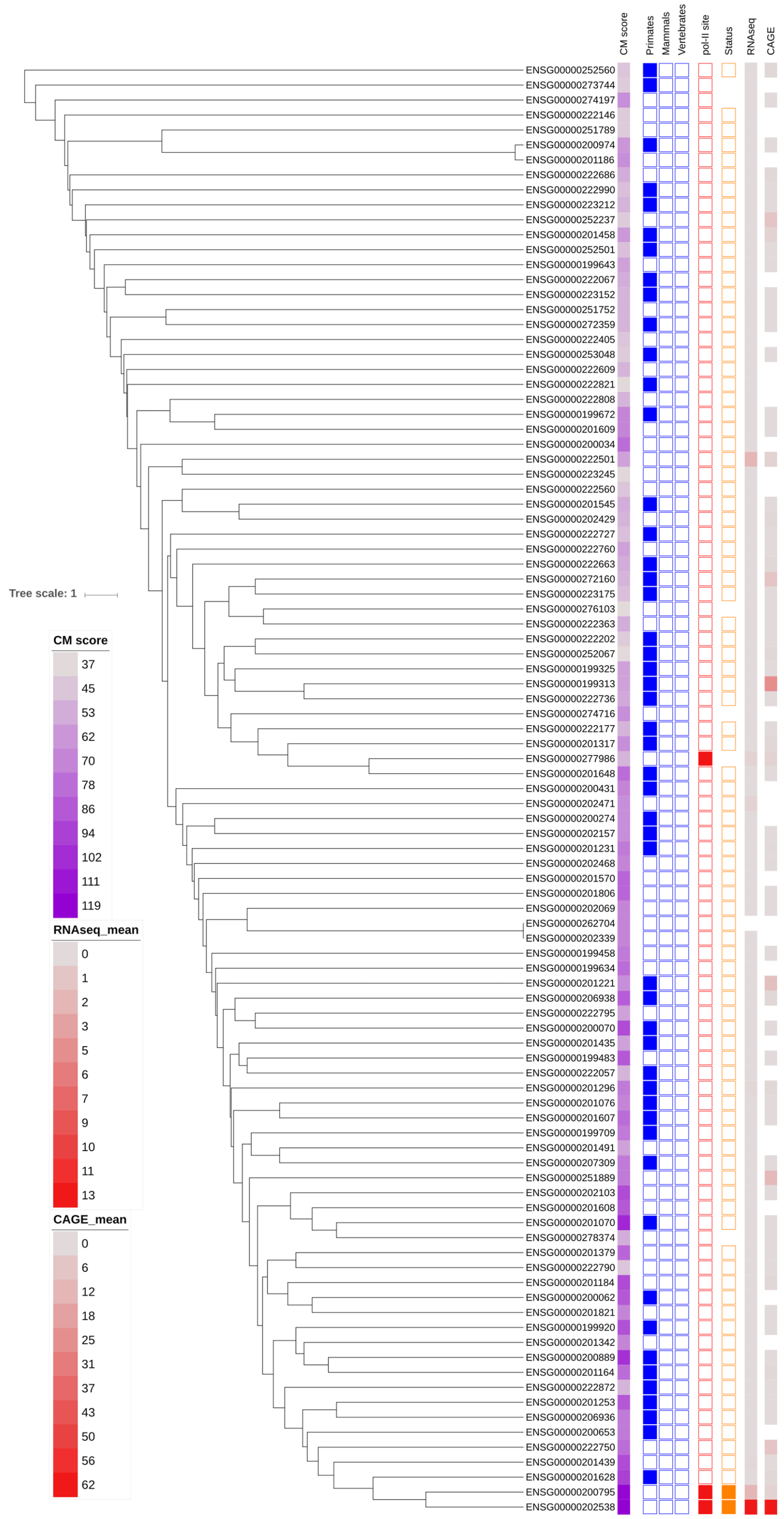

Figure S5 Tree of pairwise similarities of human U4 snRNA copies with (from left to right): 1) CM score (purple), conservation of locus in 2) primates, 3) mammals or 4) vertebrates, 5) presence of pol-II promoter upstream of locus, 6) status of gene locus in HGNC (solid = known, empty = known pseudogene, missing = not listed in HGNC) and mean expression from 7) ENCODE small RNAseq and 8) CAGE data (see Methods). Missing expression data =

gene not on primary assembly or no unambiguous call was possible to due redundant mappings.

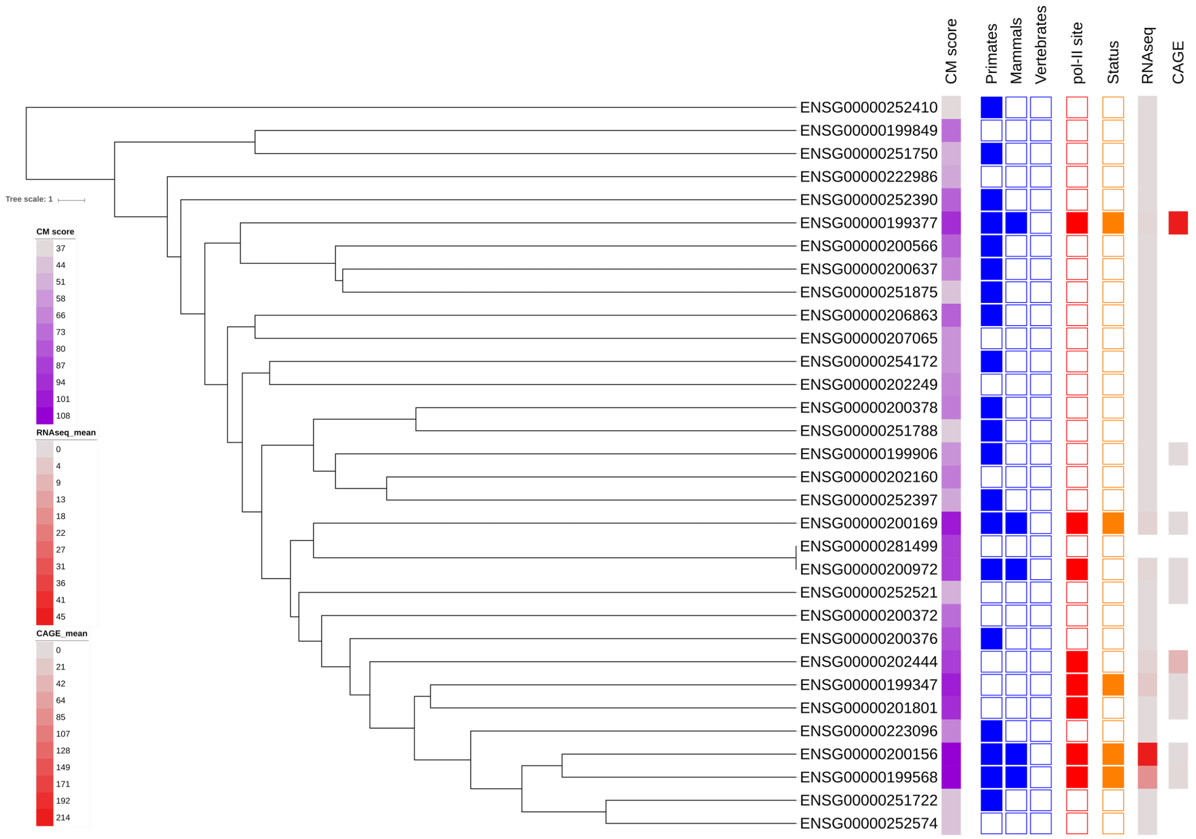

Figure S6: Tree of pairwise similarities of human U5 snRNA copies with (from left to right): 1) CM score (purple), conservation of locus in 2) primates, 3) mammals or 4) vertebrates, 5) presence of pol-II promoter upstream of locus, 6) status of gene locus in HGNC (solid = known, empty = known pseudogene, missing = not listed in HGNC) and mean expression from 7) ENCODE small RNAseq and 8) CAGE data (see Methods). Missing expression data = gene not on primary assembly or no unambiguous call was possible to due redundant mappings.

Due to the large number of loci in this family, this data cannot be visualized in the available space but is instead included as a separate vector graphics file (Figure_S7.svg).

Figure S7: Tree of pairwise similarities of human U6 snRNA copies with (from left to right): 1) CM score (purple), conservation of locus in 2) primates, 3) mammals or 4) vertebrates, 5) presence of pol-II promoter upstream of locus, 6) status of gene locus in HGNC (solid = known, empty = known pseudogene, missing = not listed in HGNC) and mean expression from) ENCODE small RNAseq and 8) CAGE data (see Methods). Missing expression data = gene not on primary assembly or no unambiguous call was possible to due redundant mappings.

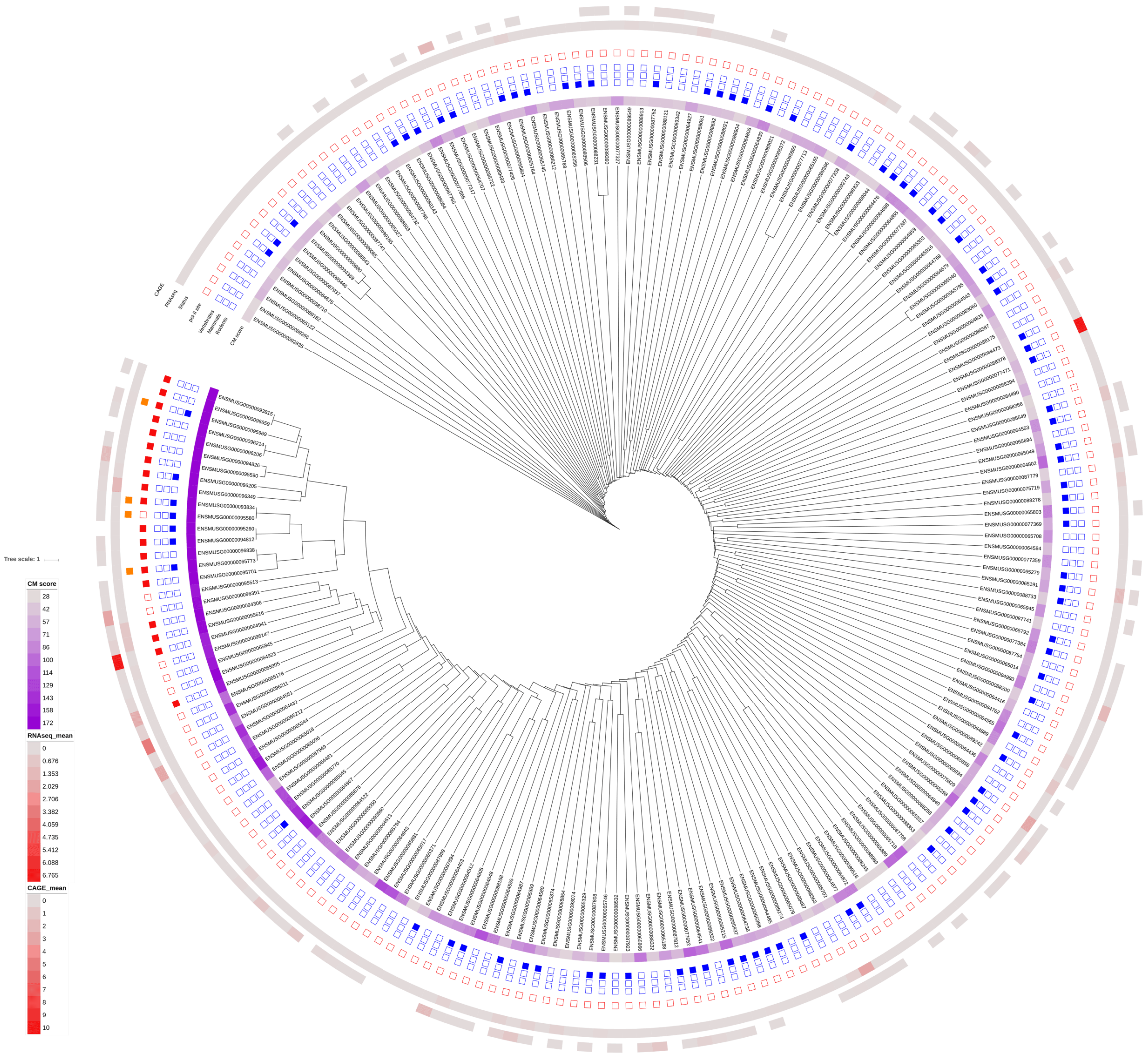

Figure S8: Tree of pairwise similarities of mouse U1 snRNA copies with (from left to right): 1) CM score (purple), conservation of locus in 2) rodents, 3) mammals or 4) vertebrates, 5) presence of pol-II promoter upstream of locus, 6) status of gene locus in MGI (solid = known, empty = predicted, missing = not listed in MGI) and mean expression from 7) ENCODE total RNAseq and 8) CAGE data (see Methods). Missing expression data = gene not on primary assembly or no unambiguous call was possible to due redundant mappings.

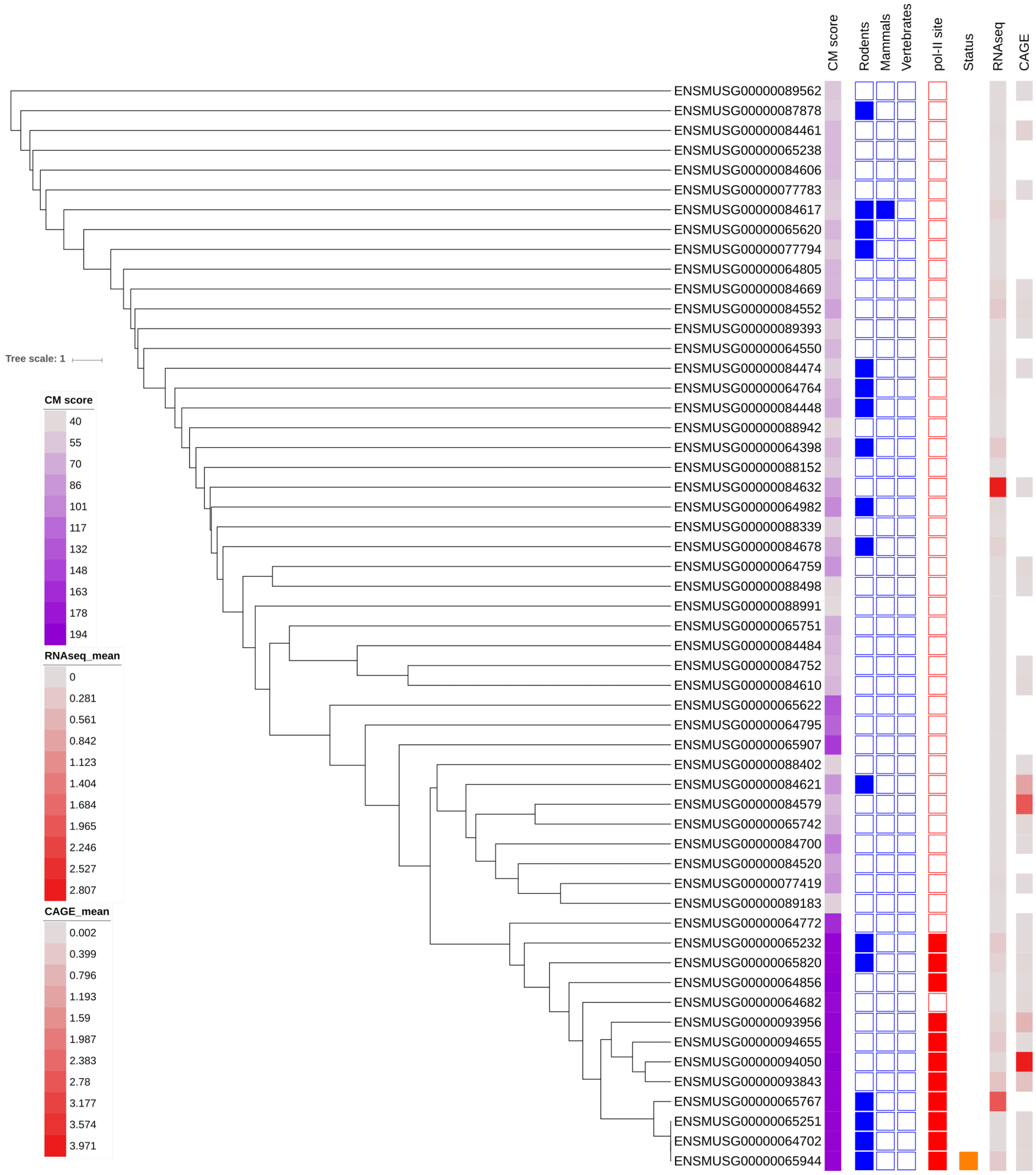

Figure S9: Tree of pairwise similarities of mouse U2 snRNA copies with (from left to right): 1) CM score (purple), conservation of locus in 2) rodents, 3) mammals or 4) vertebrates, 5) presence of pol-II promoter upstream of locus, 6) status of gene locus in MGI (solid = known, empty = predicted, missing = not listed in MGI) and mean expression from 7) ENCODE total RNAseq and 8) CAGE data (see Methods). Missing expression data = gene not on primary assembly or no unambiguous call was possible to due redundant mappings.

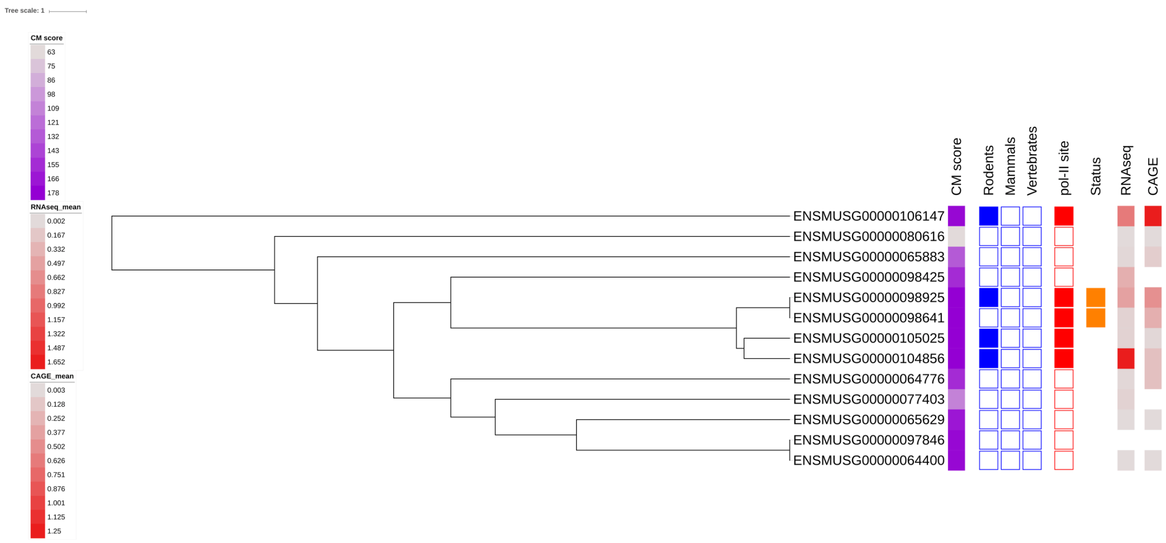

Figure S10 Tree of pairwise similarities of mouse U3 snoRNA copies with (from left to right): 1) CM score (purple), conservation of locus in 2) rodents, 3) mammals or 4) vertebrates, 5) presence of pol-II promoter upstream of locus, 6) status of gene locus in MGI (solid = known, empty = predicted, missing = not listed in MGI) and mean expression from 7) ENCODE total RNAseq and 8) CAGE data (see Methods). Missing expression data = gene not on primary assembly or no unambiguous call was possible to due redundant mappings.

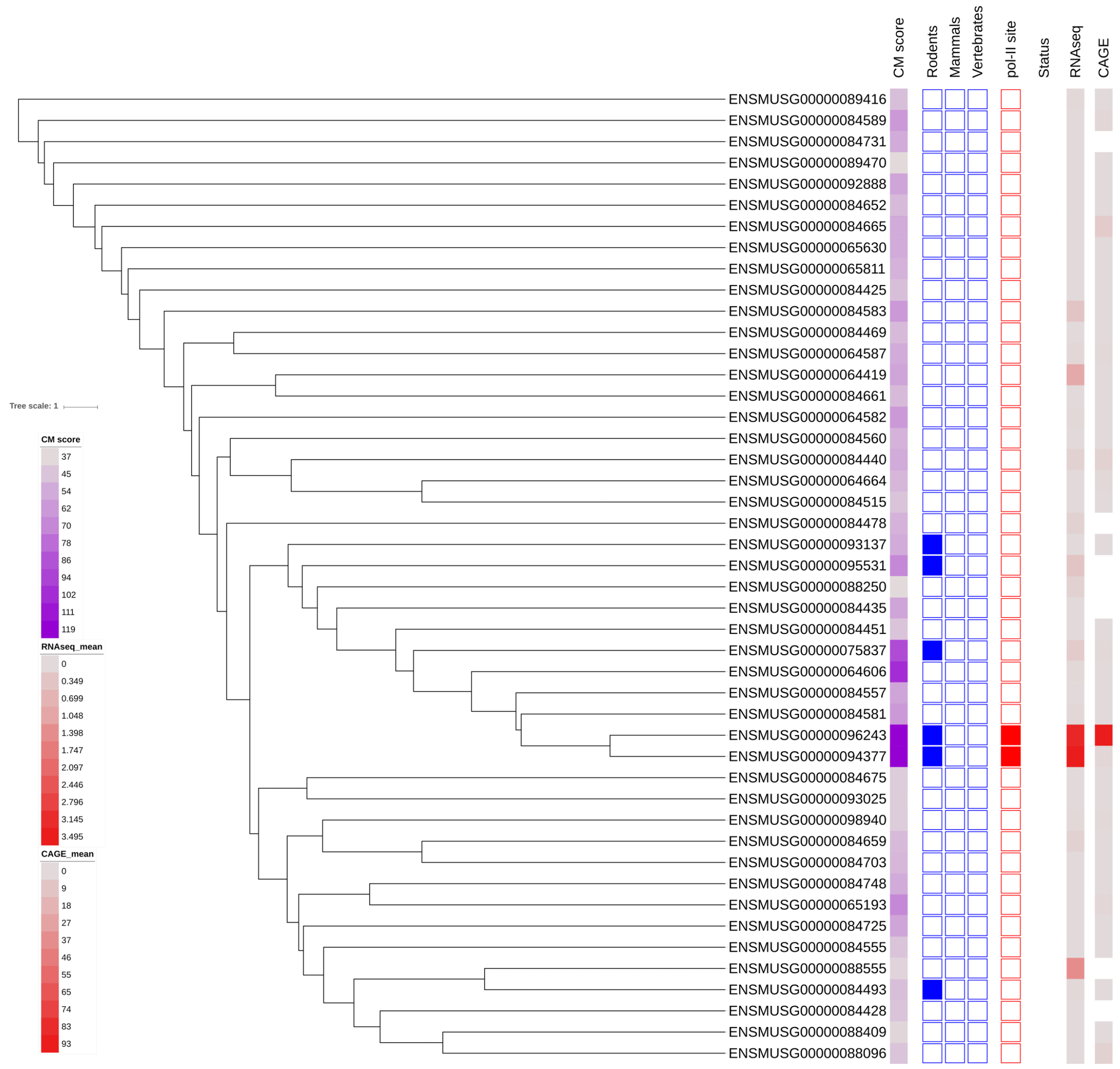

Figure S11: Tree of pairwise similarities of mouse U4 snRNA copies with (from left to right): 1) CM score (purple), conservation of locus in 2) rodents, 3) mammals or 4) vertebrates, 5) presence of pol-II promoter upstream of locus, 6) status of gene locus in MGI (solid = known, empty = predicted, missing = not listed in MGI) and mean expression from 7) ENCODE total RNAseq and 8) CAGE data (see Methods). Missing expression data = gene not on primary assembly or no unambiguous call was possible to due redundant mappings.

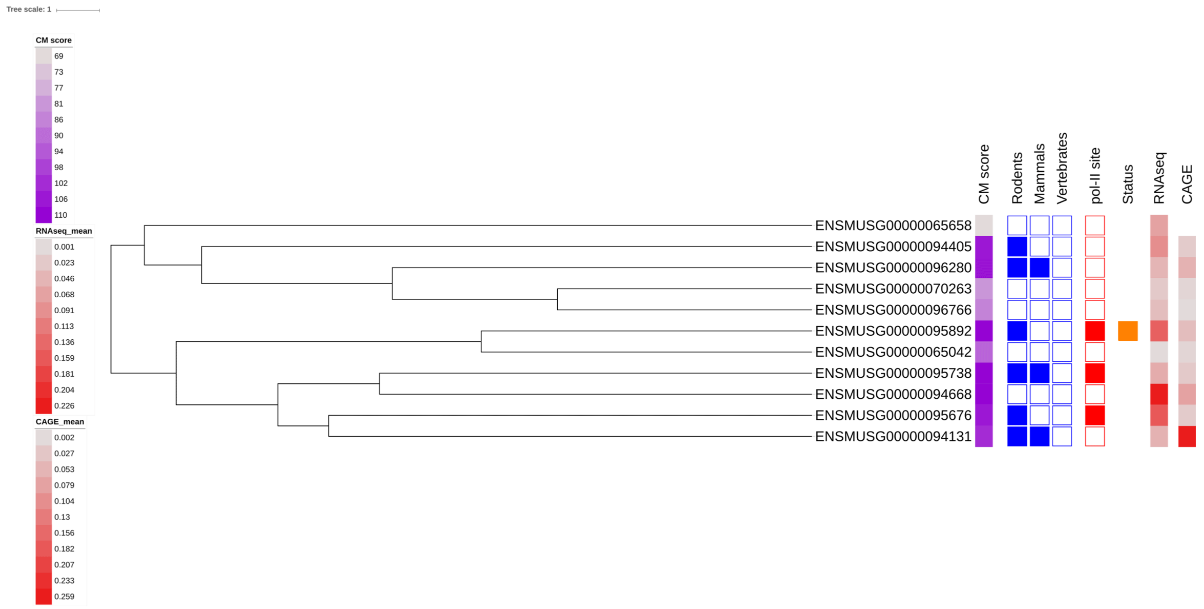

Figure S12: Tree of pairwise similarities of mouse U5 snRNA copies with (from left to right): 1) CM score (purple), conservation of locus in 2) rodents, 3) mammals or 4) vertebrates, 5) presence of pol-II promoter upstream of locus, 6) status of gene locus in MGI (solid = known, empty = predicted, missing = not listed in MGI) and mean expression from 7) ENCODE total RNAseq and 8) CAGE data (see Methods). Missing expression data = gene not on primary assembly or no unambiguous call was possible to due redundant mappings.

Due to the large number of loci in this family, this data cannot be visualized in the available space but is instead included as a separate vector graphics file (Figure_S13.svg).

Figure S13: Tree of pairwise similarities of mouse U6 snRNA copies with (from left to right): 1) CM score (purple), conservation of locus in 2) rodents, 3) mammals or 4) vertebrates, 5) presence of pol-II promoter upstream of locus, 6) status of gene locus in MGI (solid = known, empty = predicted, missing = not listed in MGI) and mean expression from 7) ENCODE total RNAseq and 8) CAGE data (see Methods). Missing expression data = gene not on primary assembly or no unambiguous call was possible to due redundant mappings.

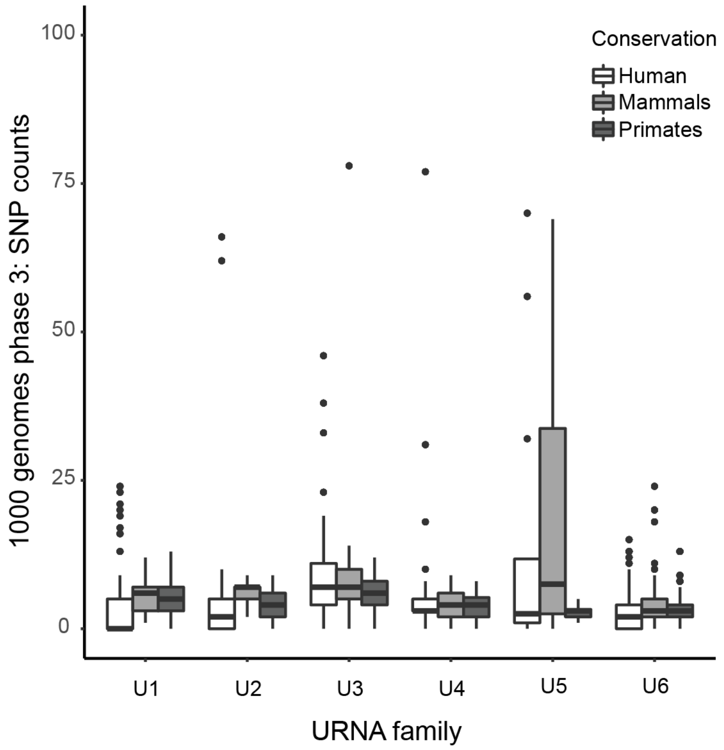

Figure S14: SNP load across URNA families in human, grouped by depth of conservation. The analysis of SNP loads from human population data shows comparable SNP loads across URNA loci irrespective of depth.

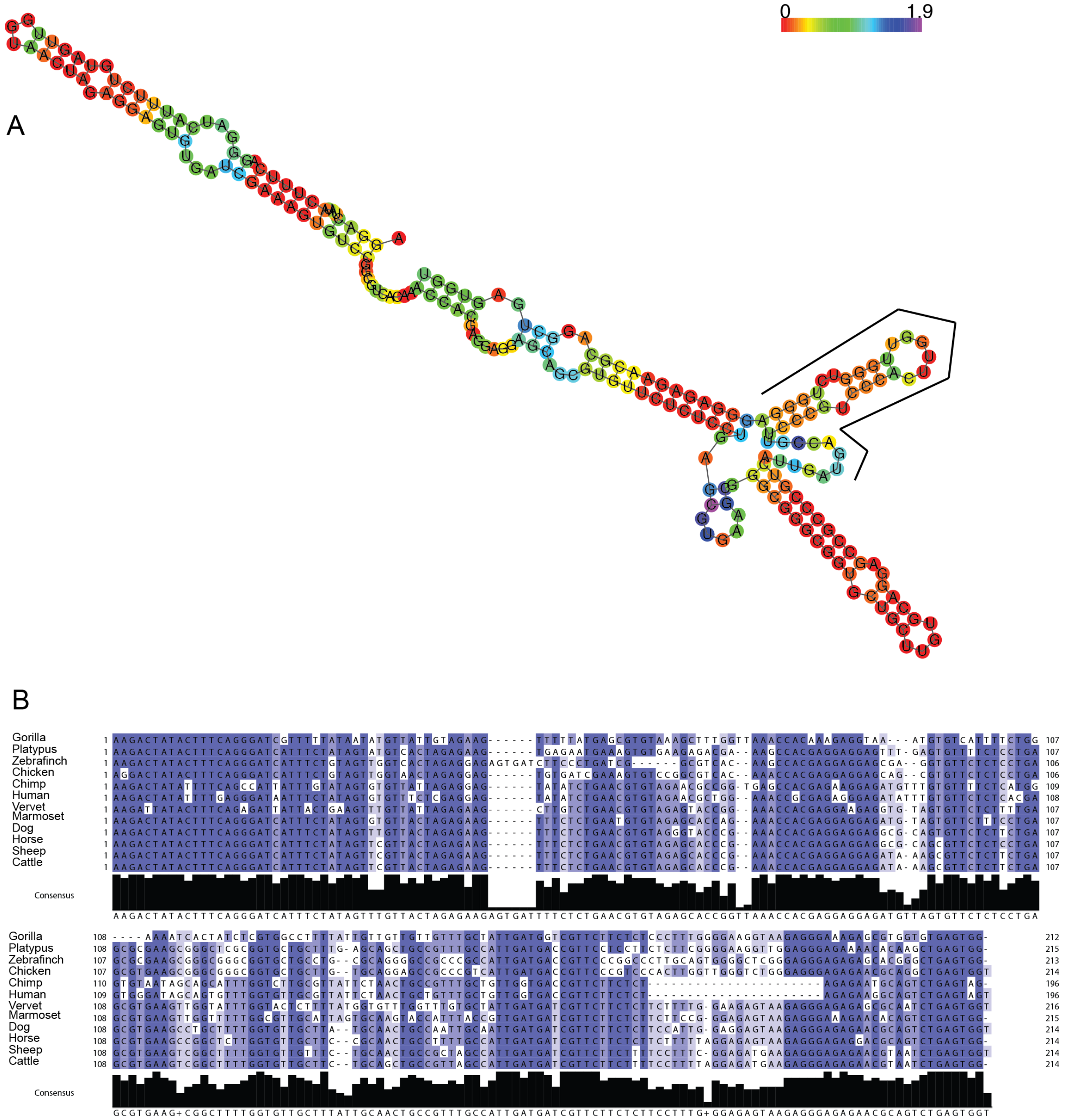

Figure S15: (A)The broadly conserved U3 locus in the testis-expressed Gene TEX14 carries a large-scale deletion (indicated by black lines) in both human and chimpanzee. Given detectable (albeit low) expression from RNA-seq data as well as the presence of an upstream pol-II promoter element, this locus may be in the process of pseudogenization or indeed the development of other functions. (B) Nucleotide sequence alignment of a deeply conserved U3 locus in the intron of the testis-expressed gene TEX14, showing a sizable deletion in the human and chimpanzee copies.

Supplementary Table S1: Conservation and genomic context of human URNA loci

			**Tracable to ancestor of**					
	**Total loci identified in human genome**		**Primates**		**Mammals**		**Vertebrates**	
	Intergenic	*Intronic*	*Intergenic*	*Intronic*	*Intergenic*	*Intronic*	*Intergenic*	*Intronic*
**U1**	93	85	17	23	0	0	0	0
**U2**	36	55	9	27	0	2	0	0
**U3**	37	26	6	14	0	1	0	1
**U4**	41	56	15	35	0	0	0	0
**U5**	14	18	7	13	2	3	0	0
**U6**	580	817	168	464	11	38	0	0

Supplementary Table S14: ENCODE human smallRNA-seq data sets

ENCODE accession number
ENCFF073RGX
ENCFF079FVD
ENCFF087EUE
ENCFF088PTQ
ENCFF094PWC
ENCFF096DFL
ENCFF102CYC
ENCFF130ZBC
ENCFF149UPH
ENCFF151VUG
ENCFF327IXJ
ENCFF349IAF
ENCFF372LLN
ENCFF456WPB
ENCFF470IXT
ENCFF486DIS
ENCFF504YKU
ENCFF505MBD
ENCFF515ENF
ENCFF519OSJ
ENCFF571MWI
ENCFF586YSA
ENCFF628ABP
ENCFF632LTU
ENCFF668WDZ
ENCFF683ZDJ
ENCFF689CZI
ENCFF692VQN
ENCFF704RNF
ENCFF707GSP
ENCFF731SJZ
ENCFF777XPZ
ENCFF779GVO
ENCFF785RWT
ENCFF799SRR
ENCFF802BSL
ENCFF820BWI
ENCFF824HJD
ENCFF881FED
ENCFF895NVP
ENCFF902FJY
ENCFF925KAH
ENCFF986EDF
ENCFF987SHZ
ENCFF988BHN

Supplementary Table S15: ENCODE mouse totalRNA-seq data sets

ENCODE accession number
ENCFF007SHF
ENCFF014ITK
ENCFF016KLR
ENCFF032XDZ
ENCFF051VXS
ENCFF052VGB
ENCFF053CRD
ENCFF059FUK
ENCFF060NTC
ENCFF062HAD
ENCFF063LXW
ENCFF070OPW
ENCFF071QDU
ENCFF082DGE
ENCFF085MBO
ENCFF093YSD
ENCFF099UUS
ENCFF101BTX
ENCFF136TII
ENCFF143NRY
ENCFF156BSL
ENCFF160LUK
ENCFF167NXN
ENCFF179JEC
ENCFF198HBZ
ENCFF216XBD
ENCFF220DJN
ENCFF229IFK
ENCFF236BUE
ENCFF237SXT
ENCFF241GLU
ENCFF249ZZI
ENCFF251LNG
ENCFF262CIY
ENCFF275SGM
ENCFF282QML
ENCFF291NWK
ENCFF300QQW
ENCFF301EKO
ENCFF327YJQ
ENCFF329ACL
ENCFF348BYM
ENCFF360NRX
ENCFF360XMZ
ENCFF367OPA
ENCFF377BWR

ENCFF378CNA
ENCFF405TRT
ENCFF405VKS
ENCFF409ZNA
ENCFF416EWW
ENCFF418MSC
ENCFF421QJA
ENCFF445AIZ
ENCFF453JXA
ENCFF477PIC
ENCFF478ZKL
ENCFF479HKB
ENCFF489RVW
ENCFF490QEC
ENCFF494GEO
ENCFF499UQZ
ENCFF500GLW
ENCFF503BOB
ENCFF503TOH
ENCFF510RXX
ENCFF526QHV
ENCFF528JFG
ENCFF528UUE
ENCFF530SXN
ENCFF536HIT
ENCFF548GUA
ENCFF551UCM
ENCFF557LMN
ENCFF562GFS
ENCFF565KTC
ENCFF576MIX
ENCFF599OTY
ENCFF635VBU
ENCFF635YMK
ENCFF638TAT
ENCFF642EVR
ENCFF646JUX
ENCFF657LQI
ENCFF671GFL
ENCFF676BDY
ENCFF677ULG
ENCFF678XFK
ENCFF679RDZ
ENCFF694UNH
ENCFF706XOL
ENCFF718DNJ
ENCFF728LAM
ENCFF738FUB
ENCFF739QUZ

ENCFF744XBD
ENCFF758NAG
ENCFF771GDS
ENCFF775HDI
ENCFF778KZE
ENCFF781WVF
ENCFF786VDJ
ENCFF798JOI
ENCFF800SJE
ENCFF807MPL
ENCFF810MMJ
ENCFF810ZDM
ENCFF819ZTA
ENCFF834WRE
ENCFF839MMS
ENCFF839UKS
ENCFF867HND
ENCFF874AXO
ENCFF876NSY
ENCFF891HIX
ENCFF896COV
ENCFF896QPV
ENCFF910RNP
ENCFF913XMQ
ENCFF916XIQ
ENCFF919VVI
ENCFF926BFE
ENCFF929PSZ
ENCFF932YNB
ENCFF956HCY
ENCFF957SPL
ENCFF959ZAX
ENCFF985XAR
