# Supplementary figures and images for: An Evaluation of Function of Multicopy Noncoding RNAs in Mammals Using ENCODE/FANTOM Data and Comparative Genomics
Source: Mol Biol Evol. 2018 Apr 3;35(6):1451–62. doi: 10.1093/molbev/msy046 (PMC5967550; doi:10.1093/molbev/msy046)

Tree scale: 1 

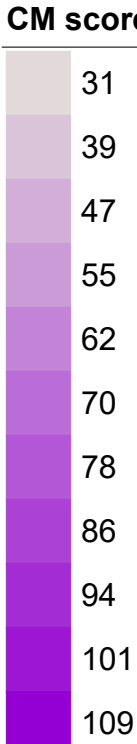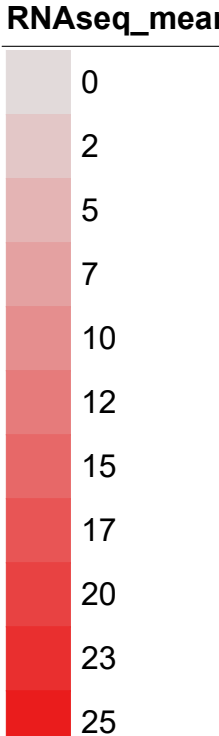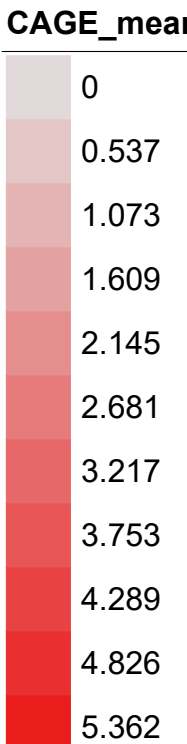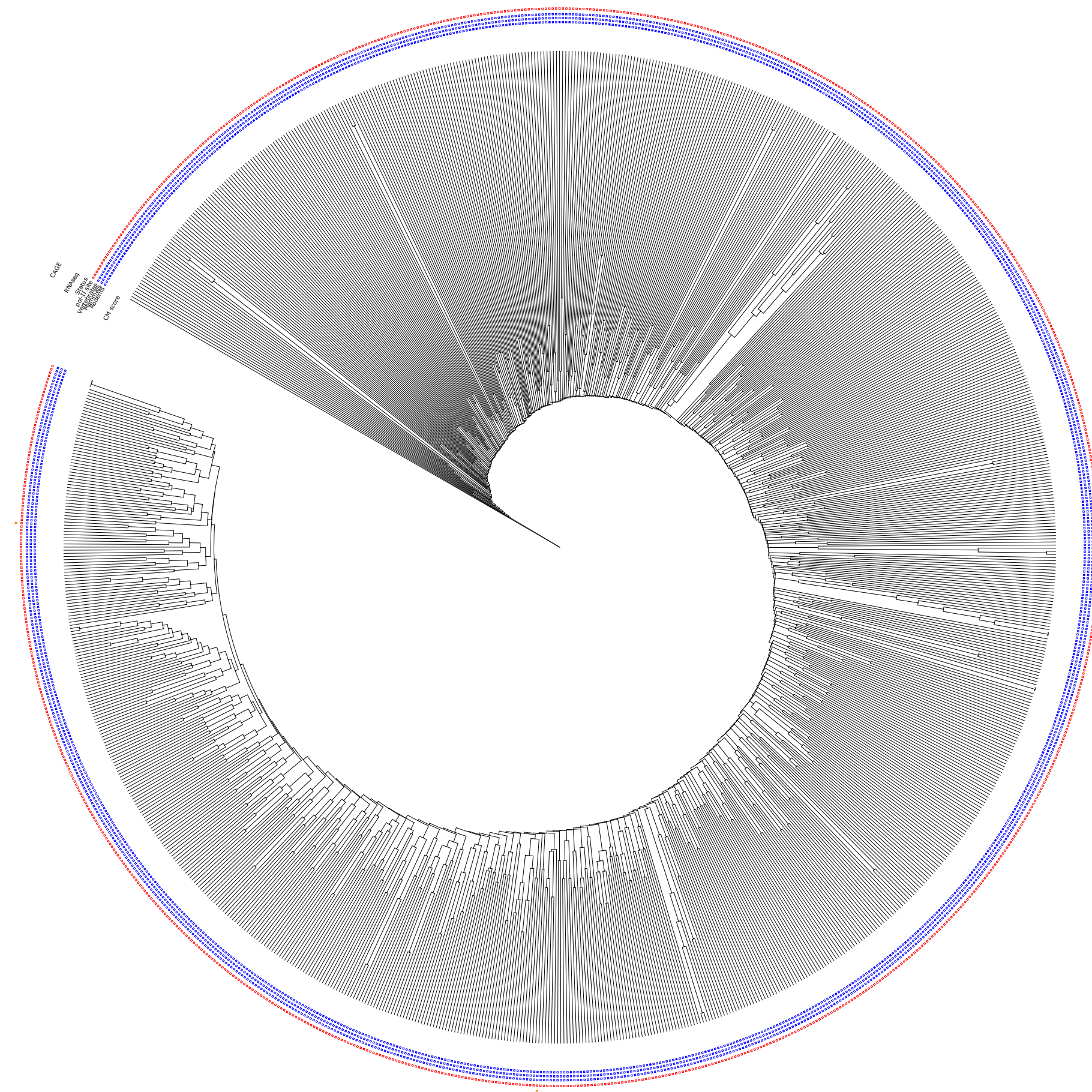

Supplement: Supplementary Data [file msy046_supp.zip › Figure_S13.pdf]

Tree scale: 1

CM score

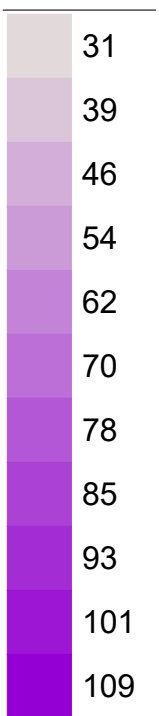

RNAseq\_mean

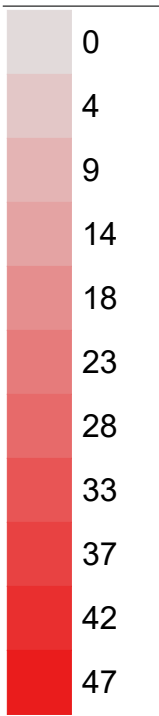

CAGE\_mean

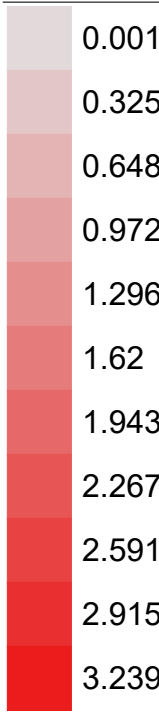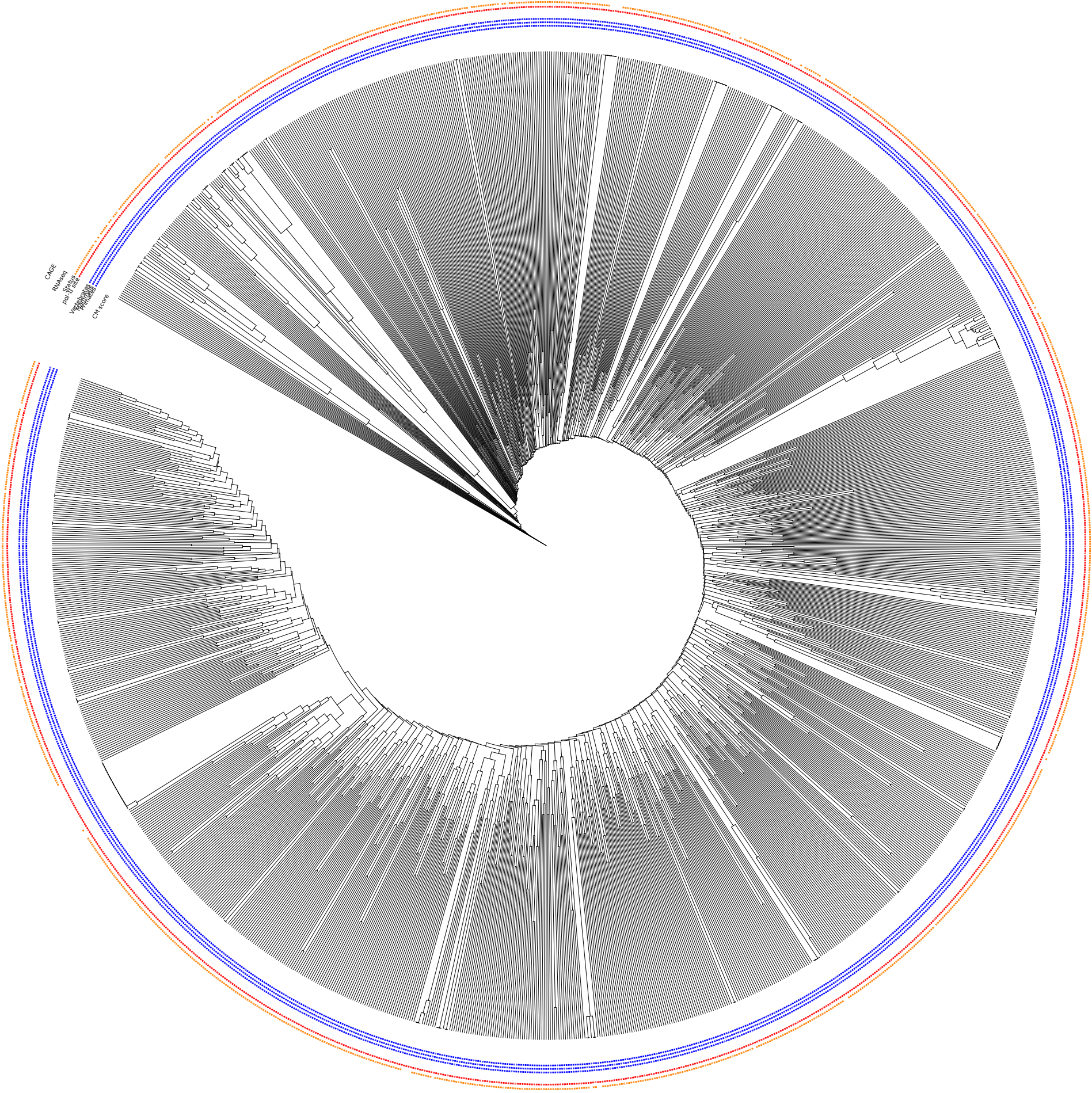

Supplement: Supplementary Data [file msy046_supp.zip › Figure_S7.pdf]
